# Transcriptome analysis revealed lncRNA-mRNA modules responsive to low temperature stress in Qingke

**DOI:** 10.1186/s12864-026-12838-0

**Published:** 2026-05-11

**Authors:** Mingzhai Yu, Deqing Zhuoga, Dabin Zhuang, Wenbo Wei

**Affiliations:** 1 Institute of Agricultural Sciences, Xizang Academy of Agriculture and Animal Husbandry Sciences, Lhasa, Xizang 850002 China; 2 State Key Laboratory of Hulless Barley and Yak Germplasm Resources and Genetic Improvement, Lhasa, Xizang 850002 China

**Keywords:** Qingke, low temperature stress, lncRNA, Photosynthesis, Antioxidant defense

## Abstract

**Background:**

Qingke (hull-less barley), a staple crop on the Qinghai-Xizang Plateau, has evolved unique stress tolerance mechanisms. Cold stress damages plants through ice formation and oxidative stress, triggering antioxidant systems and stress-related gene expression. Long non-coding RNAs (lncRNAs) are known to regulate these responses.

**Results:**

Physiological analysis revealed a dynamic defense strategy: antioxidant systems (SOD/POD) activated early (12 h), shifting to osmotic regulation (soluble protein) later (48 h). Membrane damage (MDA/conductivity) increased under stress but showed repair during recovery.

Transcriptome profiling identified a core set of 317 differentially expressed genes enriched in catalytic and stress-response functions. Early stress (12h) predominantly involved hormone (jasmonate) and pathogen defense pathways. Later (48h), protein synthesis (ribosome) and secondary metabolism (phenylpropanoid) were upregulated. Recovery (24h) activated carbon metabolism and fatty acid degradation.

Furthermore, specific lncRNAs (e.g., LNC_003210) were identified as potential regulators of photosynthesis-related genes.

**Conclusions:**

Qingke adapts to cold through phased physiological responses (antioxidant defense, osmoregulation) and dynamic metabolic reprogramming. LNC_003210 may play a key role in cold adaptation by regulating photosynthesis.

**Supplementary Information:**

The online version contains supplementary material available at 10.1186/s12864-026-12838-0.

## Introduction

Qingke (*Hordeum vulgare* L. var. *nudum* Hook.f.) is an annual herbaceous plant belonging to the Gramineae family. Due to its caryopsis being prone to detaching from the lemma upon maturity, it is also known as naked barley. Qingke is well-suited to growth in the cool climates of the plateaus and is widely distributed in northwestern China and the southwestern provinces. It is one of the primary crops in the Tibetan Plateau [1, 2]. Qingke grains are high in protein, fiber, and vitamins, and low in fat and sugar, making them highly nutritious and medicinal. In addition, Qingke serves as a raw material in the wine industry, food processing, and animal husbandry [3–5]. Since Qingke mainly grows in harsh natural environments with low temperatures and low oxygen levels, it has developed strong resistance to adverse conditions such as low temperatures, drought, and salinity through long-term natural selection and evolution [1]. As a result, Qingke has a unique stress-tolerance system and a large number of key genes that respond to adverse conditions [6]. However, in recent years, extreme weather events have become more frequent worldwide, and freeze-thaw disasters continue to threaten Qingke’s yield and quality.

Low-temperature stress is one of the world’s major disasters. When plants are exposed to low temperatures during their growing season, it can cause severe damage to plant growth and even lead to plant death [7]. The primary causes of plant damage due to low temperatures include internal freezing and membrane damage. Ice crystals larger than proteins can disrupt the structure of biological membranes, organelles, and cytoplasm, thereby causing fatal damage to cells [8]. Additionally, excessive dehydration of protoplasm at low temperatures can disrupt the molecular structure of proteins, further impairing normal cellular metabolism in plants [9–11]. Additionally, at low temperatures, the degree of lipid peroxidation in membranes increases, Malondialdehyde (MDA) levels rise, and a large amount of reactive oxygen species (ROS) are produced, such as hydrogen peroxide (H₂O₂), superoxide radicals (O₂⁻), and hydroxyl radicals (OH⁻), thereby activating the plant’s antioxidant system to eliminate reactive oxygen species, including peroxidase (POD), superoxide dismutase (SOD), and catalase (CAT), etc. In addition, under low-temperature stress, the photosynthetic capacity of plants weakens, and metabolic balance is disrupted, leading to the accumulation of metabolites such as betaine, sugars, and amino acids in plant cells to counteract the harmful effects of stress [12–15]. After plants are subjected to low-temperature stress, the transcription of related drought-tolerant genes accelerates, including heat shock proteins (HSP), osmoregulatory-related proteins, late embryogenesis abundant (LEA), and antioxidant enzyme genes [10, 16, 17].

Long non-coding RNAs (lncRNAs) are a class of RNAs no longer than 200 base pairs that cannot encode proteins [18]. LncRNA functions primarily by directly or indirectly influencing mRNA. Their effects are achieved mainly by modulating chromatin conformation to regulate cis-gene expression within chromosomal neighborhoods and downstream regions, or by trans-acting on distant genes through base-pairing with other RNAs, protein interactions, or DNA interactions that spatially bring distant genomic locations into proximity [19]. lncRNAs have been widely observed in plant responses to cold stress. *Populus simonii* responds to cold stress by enhancing lncRNA regulation in photosynthesis and plant hormone synthesis pathways [20]. In grapevine (*Vitis vinifera* L.), differentially expressed lncRNAs under cold stress target stress-response-related genes, including CBF4 transcription factor genes, late embryogenesis abundant (LEA) protein genes, peroxisome biogenesis protein genes, and WRKY transcription factor genes [21]. Functional annotation of co-expressed genes corresponding to DE lncRNAs in tea plants responding to low temperatures indicates involvement in processes including protein folding, cellular responses to reduced oxygen levels, responses to hypoxia, binding of unfolded proteins, and responses to heat during high-temperature stress; as well as responses to cold, water transport, and aquaporin activity during low-temperature stress [22]. In *Ammopiptanthus nanus* under cold stress, 421 lncRNAs were identified as participating in cold stress responses by forming lncRNA-mRNA modules and regulating genes encoding stress-related transcription factors and enzymes via cis-acting mechanisms. while 31 lncRNAs serving as miRNA precursors and 8 lncRNAs acting as endogenous competitive targets for miRNAs participated in cold stress responses by forming lncRNA-miRNA-mRNA regulatory modules [23]. Therefore, this study used the Qingke variety Longzi purple Qingke as experimental material and employed physiological and biochemical, as well as transcriptomics techniques, to investigate the physiological and molecular mechanisms of Qingke in response to low-temperature stress. The aim was to identify key biological processes and metabolic pathways associated with cold tolerance, and to identify key genes involved in cold tolerance, thereby providing data and theoretical support for breeding cold-tolerant Qingke varieties.

## Result

### Changes in physiological indicators of hull barrey (Qingke) exposed to 0 ℃ for varying durations

**Fig. 1:**
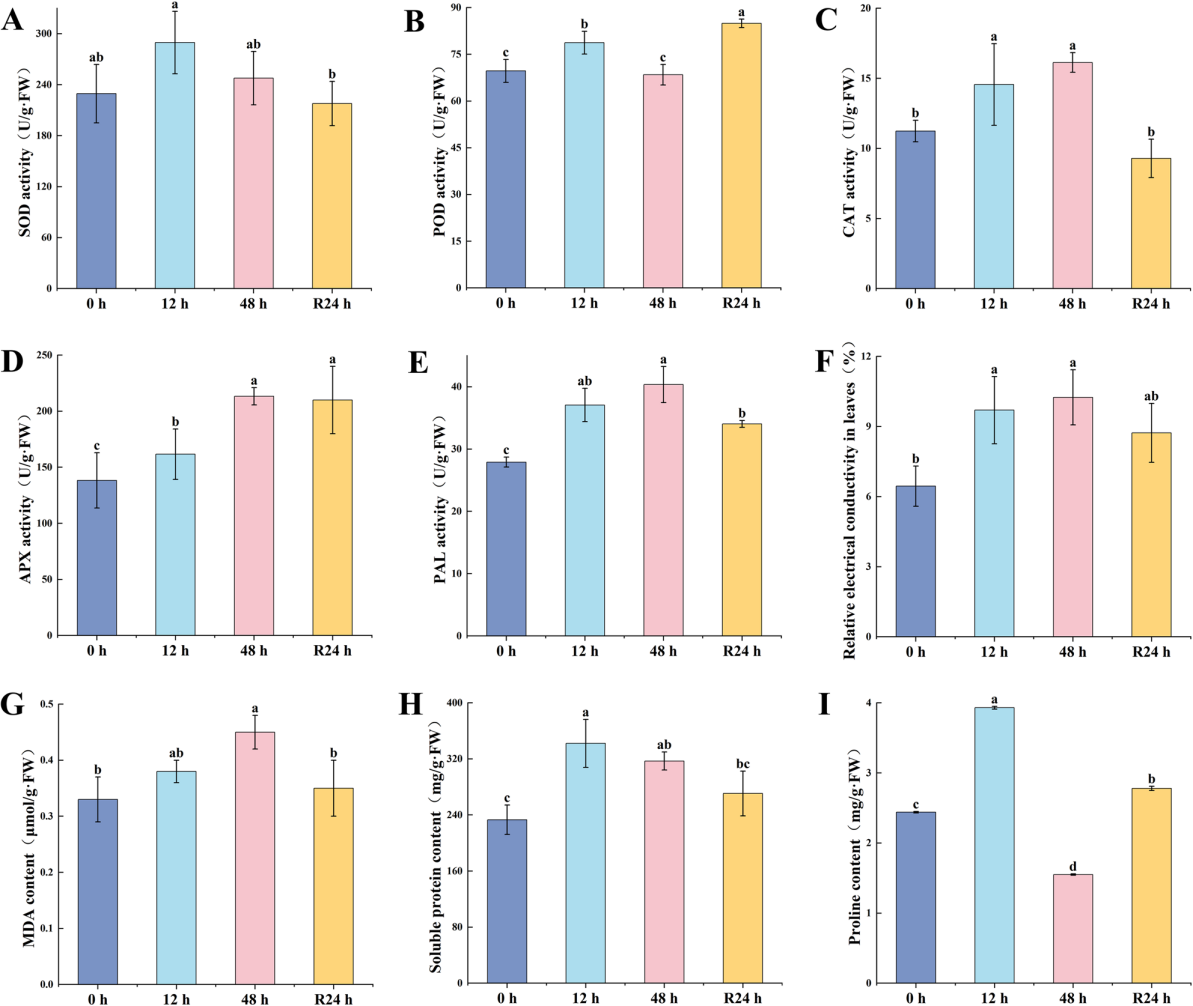
Changes in Qingke’s physiological indicators at 0℃ under different treatment durations. Note: **A** represents SOD, **B** represents POD, **C** represents CAT, **D** represents APX, **E** represents PAL, **F** represents relative electrical conductivity, **G** represents MDA, **H** represents soluble protein, and **I** represents proline

To determine the effects of different treatment durations at 0℃ on Qingke, we analyzed physiological indicators for four treatments: 0 h (CK), 12 h, 48 h, and 24 h recovery at 24 °C (Re 24 h). SOD (Fig. 1A) showed an initial increase followed by a decrease, continuing to decline after Re 24 h and exhibiting a slight decrease compared to CK; POD (Fig. 1B) increased with increasing treatment time before decreasing, but then showed a significant increase after Re 24 h; as treatment duration increased, CAT (Fig. 1C), Ascorbate peroxidase(APX)(Fig. 1D), Phenylalanine ammonia-lyase ༈PAL༉(Fig. 1E), relative electrical conductivity༈REC༉ (Fig. 1F), MDA (Fig. 1G), and soluble protein (Fig. 1H) all increased with increasing treatment time, but decreased again after Re 24 h; proline (Fig. 1I) increased with increasing treatment time, then decreased, but increased again after Re 24 h. Proline levels significantly increased at 12 h compared to 0 h, then significantly decreased at 48 h relative to 0 h. Re24h levels were significantly higher than at 0 h and 48 h but significantly lower than at 12 h.

### Transcriptome results analysis

To study Qingke’s adaptability to cold stress, lncRNA analysis was conducted on samples collected after cold stress. The results indicated that the RNA quality control was satisfactory, with Clean Reads for the 12 tested samples all exceeding 80,789,728, and Clean Bases ranging from 12.12 to 14.52 gigabases; the error rate was 0.03%. The highest Q20 value was 97.77%, and the lowest was 97.09%; the GC content varied between 45.09 and 47.82% (refer to Supplementary Table 2). Comparative analysis with the reference genome revealed that the percentage of Reads mapped ranged from 66.64 to 81.43%, Unique mapped reads ranged from 31.60 to 46.60%, Multi mapped reads ranged from 23.45 to 46.94%, and the mapping of Read1 was similar to that of Read2, with ‘+’ mapped reads closely matching ‘-’ mapped reads (refer to Supplementary Table 3). Correlation analysis showed that all correlation coefficients were 0.96, indicating that the expression patterns across all samples were highly similar (see Supplementary Fig. 1).

### Differential mRNA analysis across different treatments

**Fig. 2:**
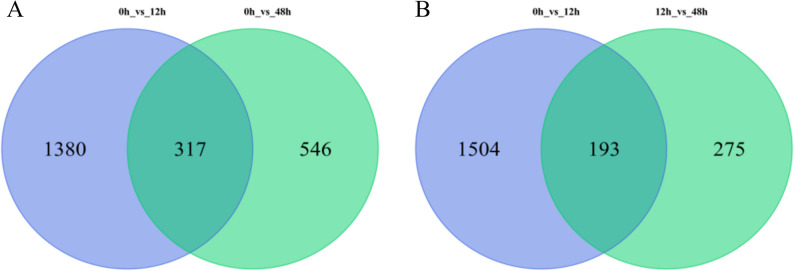
Venn diagram of differential mRNAs. Note: **A** shows the Venn diagram for 0 h vs 12 h and 0 h vs 48 h. **B** shows the Venn diagram for 0 h vs 12 h and 12 h vs 48 h

Low-temperature stress induces a significant response in many genes in Qingke. As shown in Tables 1, 1,697 differentially expressed genes were identified 12 h after low-temperature stress, among which 1,358 genes (80.02%) were downregulated, and 339 genes were upregulated. After 48 h of low-temperature stress, 863 differentially expressed genes were identified, with 554 (64.19%) genes downregulated and 309 genes upregulated. After recovery for 24 h, compared with 48 h at low temperature, a total of 831 differentially expressed genes were identified, with the proportions of up- and down-regulated genes roughly equal. Compared with 12 h of low temperature, a total of 1,112 differentially expressed genes were identified, with the number of up-regulated genes exceeding that of down-regulated genes. Venn diagram analysis showed that 317 genes were differentially expressed between 0 h and 12 h, and between 0 h and 48 h. 193 genes showed differential expression in both 0 h vs. 12 h and 12 h vs. 48 h (Fig. 2). This suggests that the genes may be key to Qingke’s response to low-temperature stress and implies that Qingke’s response patterns to low temperatures vary with stress duration.

**Table 1:** Statistics on the number of differentially expressed mRNAs

group	total	down	up
0h_vs_12h	1697	1358	339
0h_vs_48h	863	554	309
0h_vs_Re24h	1399	716	683
12h_vs_48h	468	240	228
12h_vs_Re24h	1112	422	690
48h_vs_Re24h	831	416	415

### GO enrichment analysis of differentially expressed mRNA

GO enrichment analysis of differentially expressed genes during cold stress and recovery revealed consistently enriched terms across stages. At 0h_vs_12h (Supplementary Fig. 2A), 0h_vs_48h (Supplementary Fig. 2B), and 12h_vs_48h (Supplementary Fig. 2C), DEGs were primarily enriched in Cellular Component (cell, cell part, organelle), Biological Process (cellular process, metabolic process, response to stimulus), and Molecular Function (binding, catalytic activity, transporter or structural molecule activity). These similar patterns underscore the central role of catalytic activity in cold stress response. In contrast, during 48h_vs_Re24h (Supplementary Fig. 2D), enrichment was limited to cellular and metabolic processes, with catalytic activity and binding as key functions. Qingke’s exhibits similar major biological processes during room-temperature (24℃) recovery as under low-temperature stress, further demonstrating the importance of catalytic activity in low-temperature stress responses.

### Differentially expressed mRNA KEGG enrichment analysis

**Fig. 3:**
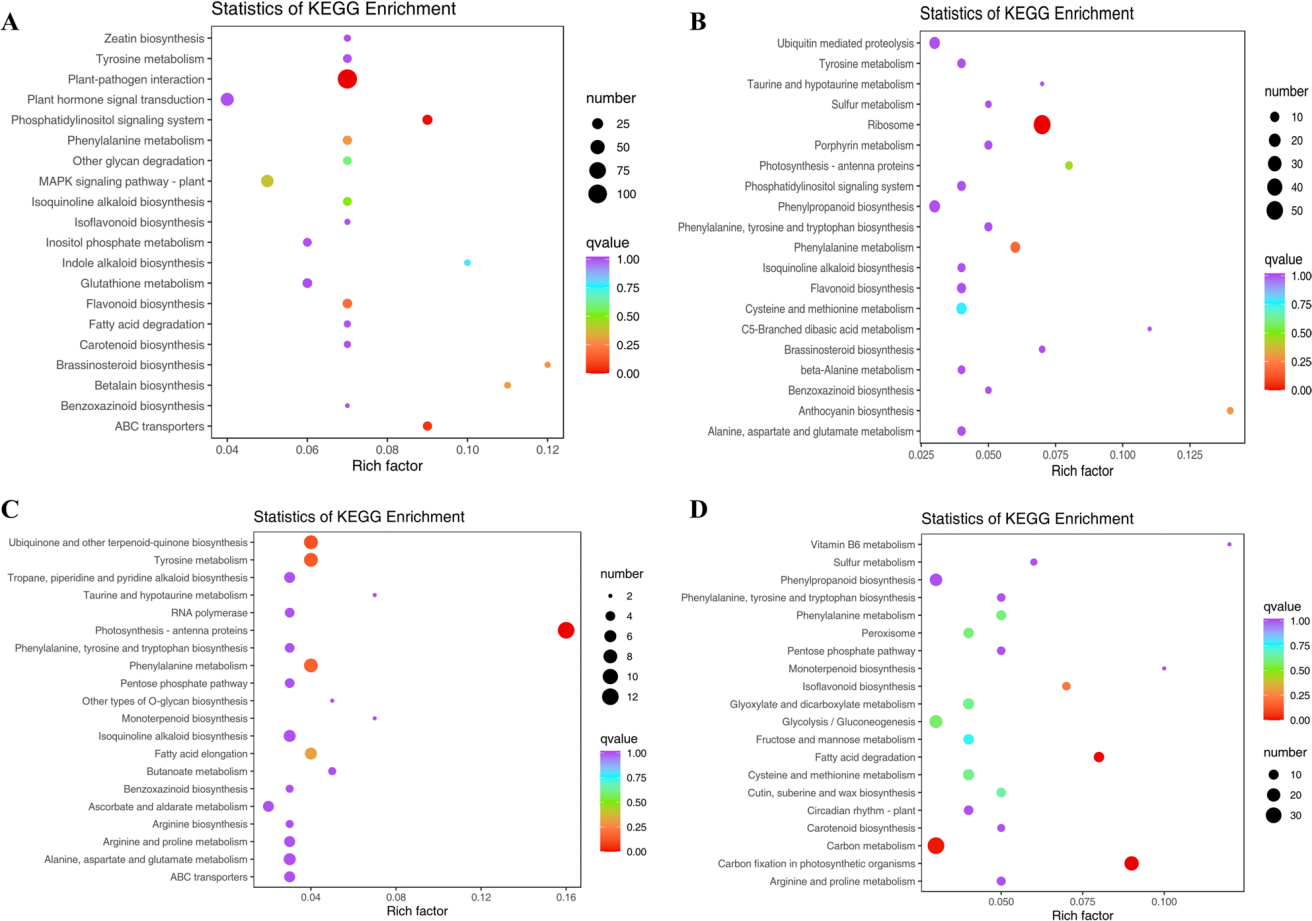
KEGG enrichment analysis of Qingke seedling leaves under low-temperature stress. Note: **A** shows the Venn diagram for 0 h vs 12 h and 0 h vs 48 h. **B** shows the Venn diagram for 12 h vs 48 h and 48 h vs Re24 h

As shown in Fig. 3, the pathways in which differentially expressed genes were mainly enriched after 12 h of low-temperature stress in Qingke included brassinosteroid biosynthesis and betalain biosynthesis. The pathways with the highest number of enriched genes were plant-pathogen interaction and plant hormone signal transduction. After 48 h of low-temperature stress, the pathways with the highest enrichment of differentially expressed genes were Anthocyanin biosynthesis and C5-Branched dibasic acid metabolism, with the pathways with the highest number of enriched genes being Ribosome, Phenylpropanoid biosynthesis, and Ubiquitin-mediated proteolysis. Compared with Re24 h at low temperature, the differentially expressed genes were primarily enriched for the Photosynthesis − antenna proteins pathway, as well as Ubiquinone and other terpenoid−quinone biosynthesis, Tyrosine metabolism, and Phenylalanine metabolism. Compared to 48 h of low temperature, the pathways enriched with differentially expressed genes after 24 h of recovery primarily included Carbon fixation in photosynthetic organisms, Carbon metabolism, and Fatty acid degradation.

### Differentially expressed lncRNA analysis

**Fig. 4:**
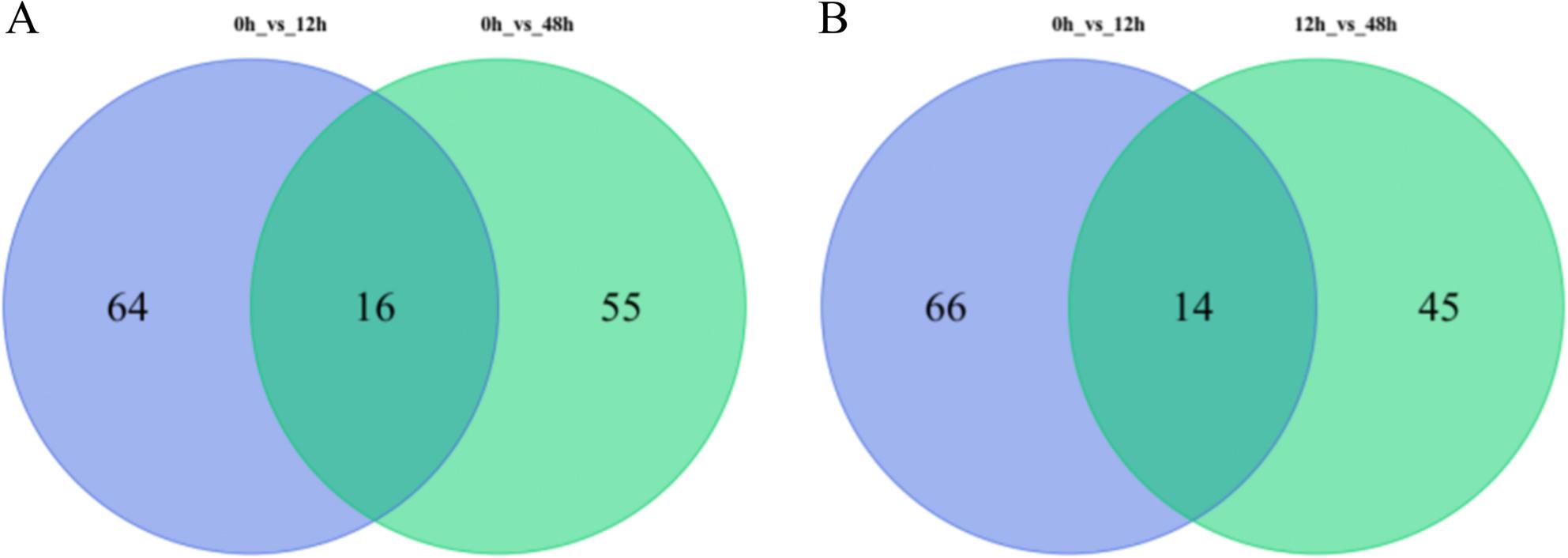
Venn diagram of differentially expressed lncRNAs. Note: **A** shows the Venn diagram for 0 h vs 12 h and 0 h vs 48 h. **B** shows the Venn diagram for 0 h vs 12 h and 12 h vs 48 h

As shown in Table 2, Qingke also produced a large number of differentially expressed lncRNAs after low-temperature stress. After 12 h of low-temperature stress, a total of 80 lncRNAs were produced, with 47 upregulated and 33 downregulated. After 48 h of low-temperature stress, a total of 71 lncRNAs were produced, with 10 downregulated and 61 upregulated, with the number of upregulated lncRNAs being six times that of downregulated lncRNAs. Compared with 48 h of low-temperature stress, 59 lncRNAs were produced after 12 h of low-temperature stress, of which 40 were downregulated, and 19 were upregulated. Compared with 48 h of low-temperature stress, 65 lncRNAs were produced after 24 h of recovery, of which 26 were downregulated, and 39 were upregulated (Table 2). The changes and proportions of differentially expressed lncRNAs in each treatment are similar to those of mRNA. Venn diagram analysis showed that 16 lncRNAs were commonly differentially expressed in 0 h vs. 12 h and 0 h vs. 48 h, and 14 lncRNAs were commonly differentially expressed in 0 h vs. 12 h and 12 h vs. 48 h, suggesting that these lncRNAs are key genes in Qingke’s response to low-temperature stress(Fig. 4).

**Table 2:** Statistics on the number of differentially expressed lncRNAs

group	total	down	up
0h_vs_12h	80	47	33
0h_vs_48h	71	10	61
0h_vs_Re24h	162	27	135
12h_vs_48h	59	40	19
12h_vs_Re24h	104	33	71
48h_vs_Re24h	65	26	39

### GO enrichment analysis of differentially expressed lncRNA target genes

Like 0h_vs_48h (Supplementary Fig. 3B), differentially expressed lncRNA target genes at 0h_vs_12h (Supplementary Fig. 3A) were primarily enriched in Biological process for electron transport chain, in Cellular component for photosystem, and in Molecular function for electron transfer activity. For 12 h vs. 48 h (Supplementary Fig. 3C), differentially expressed lncRNA target genes were primarily enriched in Biological Processes such as cellular respiration and energy derivation by oxidation of organic compounds. In the Cellular Component, enrichment was mainly observed in the proton-transporting ATP synthase complex and the proton-transporting two-sector ATPase complex. In molecular function, they were enriched in ATPase activity, coupled to movement of substances; ATPase activity, coupled to transmembrane movement of substances; and primary active transmembrane transporter activity. The target genes of differentially expressed lncRNAs in the 48h_vs_Re24h group (Supplementary Fig. 3D) showed enrichment in Biological Processes, primarily in cellular metabolic compound salvage, cellular respiration, and energy derivation by oxidation of organic compounds. In the Cellular Component category, enrichment was observed for the proton-transporting ATP synthase complex. In Molecular Function, enrichment was seen in electron transfer activity and rRNA binding.

### KEGG enrichment analysis of differentially expressed lncRNA target genes

**Fig. 5:**
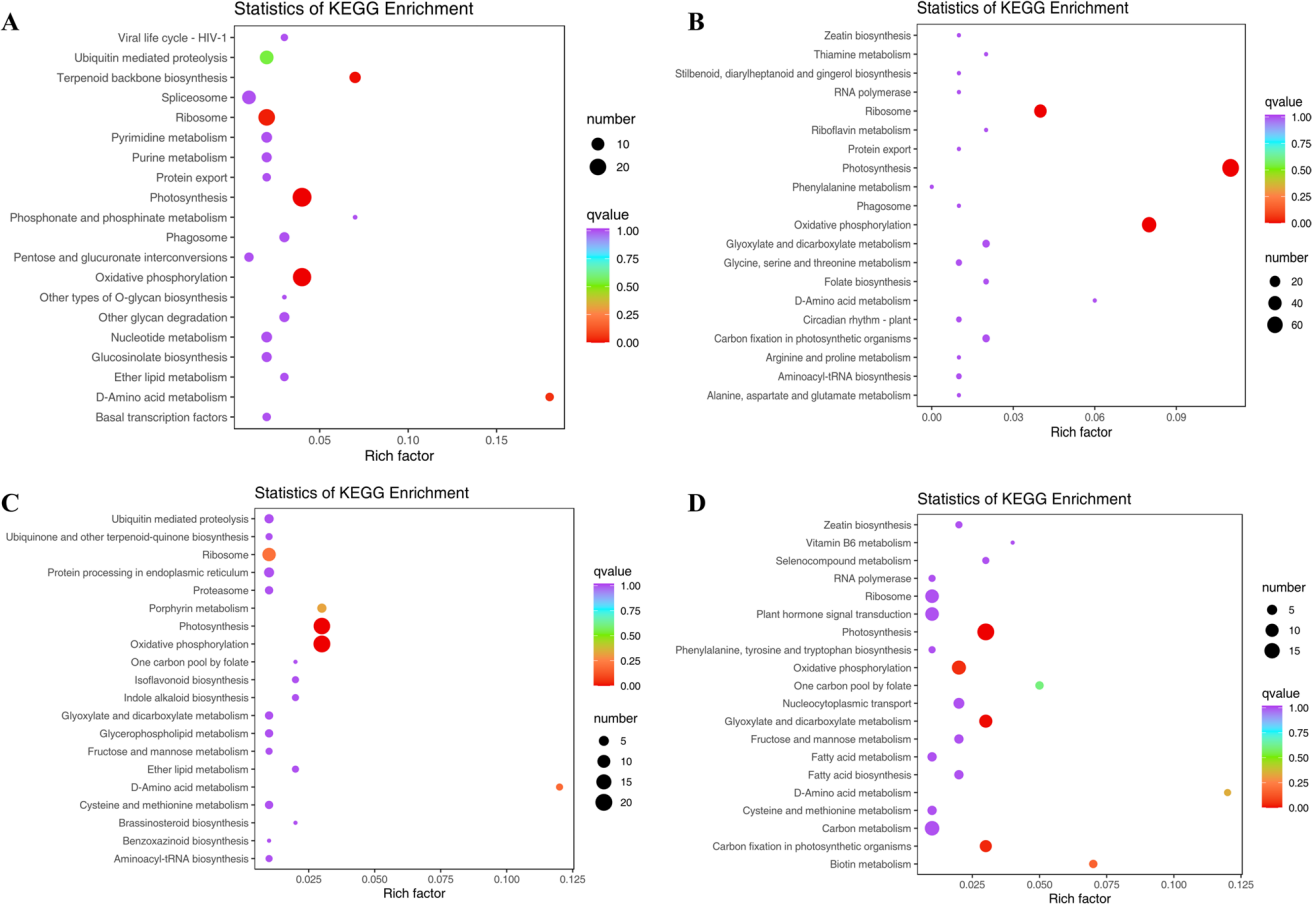
KEGG enrichment analysis of Qingke seedling leaves under low-temperature stress. Note: **A** is 0h_vs_12h, **B** is 0h_vs_48h, **C** is 12h_vs_48h, **D** is 48h_vs_Re24h

KEGG enrichment analysis of lncRNA target genes produced after low-temperature stress and recovery is shown in the figure. After 12 h of low-temperature stress, the target genes were mainly enriched in D-amino acid metabolism, with the pathways with the highest number of enriched target genes being photosynthesis and oxidative phosphorylation (Fig. 5A). After 48 h of low-temperature stress, the target genes were mainly enriched in photosynthesis, oxidative phosphorylation, and ribosomes (Fig. 5B). Compared with 12 h of low-temperature stress, the differentially expressed lncRNA target genes after 48 h of low-temperature stress were mainly enriched in D-amino acid metabolism, with the pathways with the highest number of enriched target genes being Photosynthesis and Oxidative phosphorylation (Fig. 5C). The differentially expressed lncRNA target genes after 24 h of recovery and 48 h of low-temperature stress were many enriched in D-amino acid metabolism, with Photosynthesis having the highest number of enriched target genes (Fig. 5D). Some enzymes of D-amino acid metabolism are integral to chloroplast structural integrity. The synthesis of D-alanine dimers, for example, is essential for normal chloroplast division in plants. Disrupting this pathway with inhibitors such as D-cycloserine directly causes aberrant chloroplast morphology and division failure [24, 25]. This demonstrates that a robust D-amino acid metabolic system is a prerequisite for maintaining the normal structure and function of chloroplasts. In summary, the differentially expressed lncRNA target genes under various treatments were primarily enriched for Photosynthesis and D-amino acid metabolism, indicating that this pathway is a key component of Qingke’s response to low-temperature stress.

### Changes in photosynthesis in Qingke under low-temperature stress

**Fig. 6:**
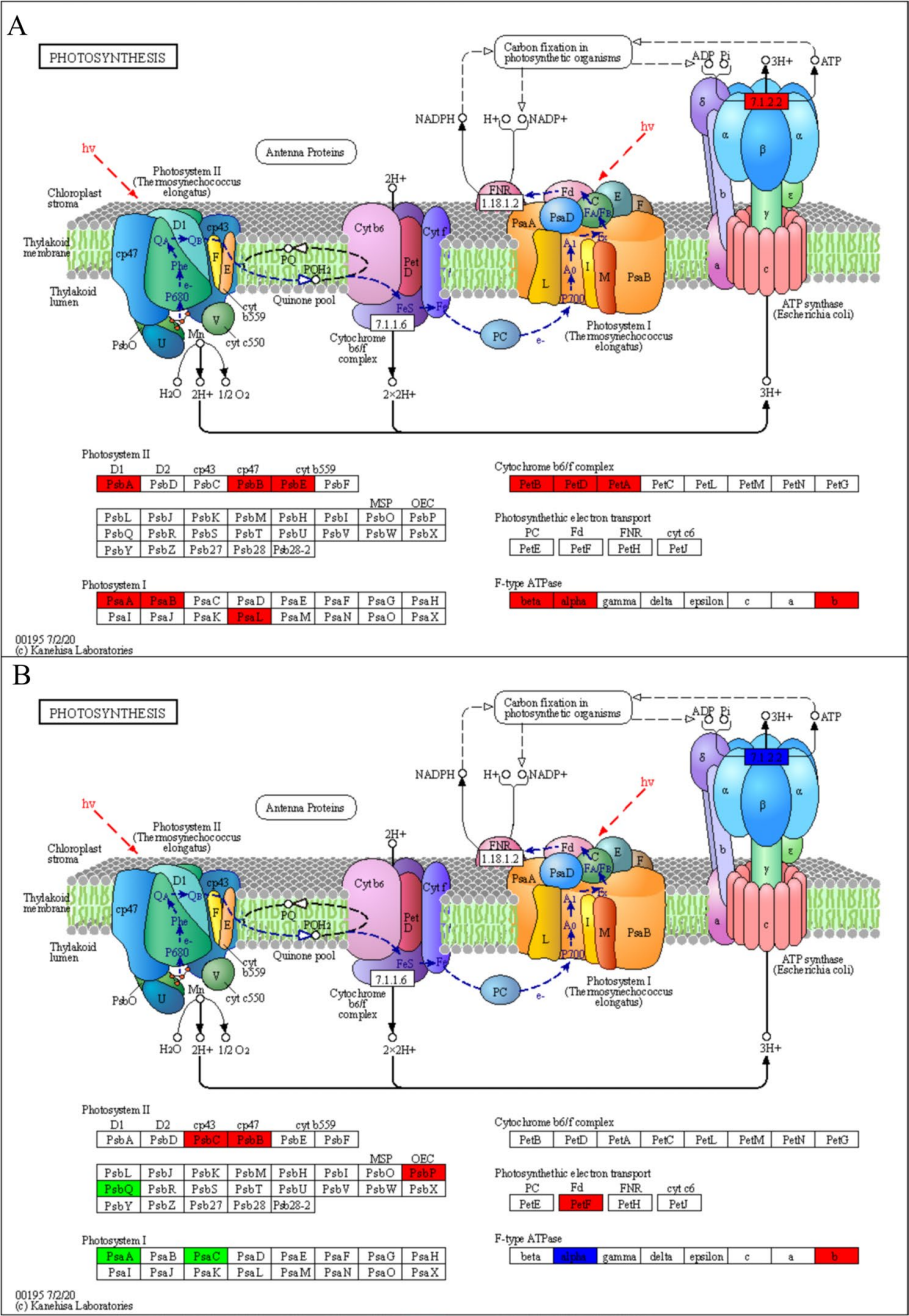
Changes in target genes of differentially expressed lncRNAs in the photosynthetic system (**A**) and changes in differentially expressed mRNAs in the photosynthetic system (**B**)

Previous studies have shown that photosynthesis plays an important role in Qingke’s response to low-temperature stress. Therefore, we further analyzed the changes in key genes in the photosynthesis pathway, as shown in Fig. 6A. All target genes of differentially expressed lncRNAs related to photosynthesis were upregulated, such as the PSbA and PSbB genes in photosystem II and photosystem I, as well as PetB, PetD, and PetA (Fig. 6B).

### lncRNA target gene prediction

LncRNAs exhibit diverse functions and can bind to DNA, RNA, and proteins to perform their roles. LncRNAs regulate protein-coding genes through their cis-regulatory relationships (based on their spatial proximity to protein-coding genes) and trans-regulatory relationships (based on their co-expression patterns), thereby fulfilling their biological functions. We set the threshold for target gene selection based on positional relationships to 100 kb upstream and downstream of lncRNA, predicting a total of 3,701 lncRNAs and 22,987 lncRNA-mRNA pairs (2,032 antisense pairs, 10,098 downstream pairs, 833 sense-overlapping pairs, and 10,023 upstream pairs). We can predict lncRNA target genes through correlation analysis or co-expression analysis of lncRNA and mRNA expression levels between samples, identifying 1,946 lncRNAs that function in a trans manner and 46,011 lncRNA-mRNA pairs. To identify differentially expressed lncRNA-mRNA pairs, the differentially expressed lncRNA target genes were intersected with the differentially expressed mRNAs. In the 0 h vs. 12 h comparison group, 80 genes were differentially expressed (47 up-regulated and 33 down-regulated). In the 0h_vs_48h comparison group, there were a total of 71 differentially expressed genes (10 up-regulated and 61 down-regulated), and a total of 162 differentially expressed genes (27 up-regulated and 135 down-regulated) in the 0h_vs_Re24h comparison group. We took the intersection of the differentially expressed lncRNAs from the three comparison groups and obtained a total of 11 lncRNAs corresponding to 75 mRNAs. KEGG functional enrichment showed that many mRNAs were enriched in chloroplast and ribosome functions, with a few enriched in the flavonoid pathway and growth-related pathways. We selected the lncRNA LNC_003210 with the highest expression level corresponding to its mRNA. NCBI BLAST analysis identified four gene IDs: OX338448.1, KC912687.1, XM_045129679, and XM_045092461. LNC_003210 shares partial sequence overlaps with OX338448.1 and KC912687.1, while exhibiting no sequence overlap with XM_045129679 or XM_045092461. This indicates that the lncRNA plays a crucial role in Qingke’s cold tolerance.

### RT-qPCR validation of lncRNA and mRNA expression

**Fig. 7:**
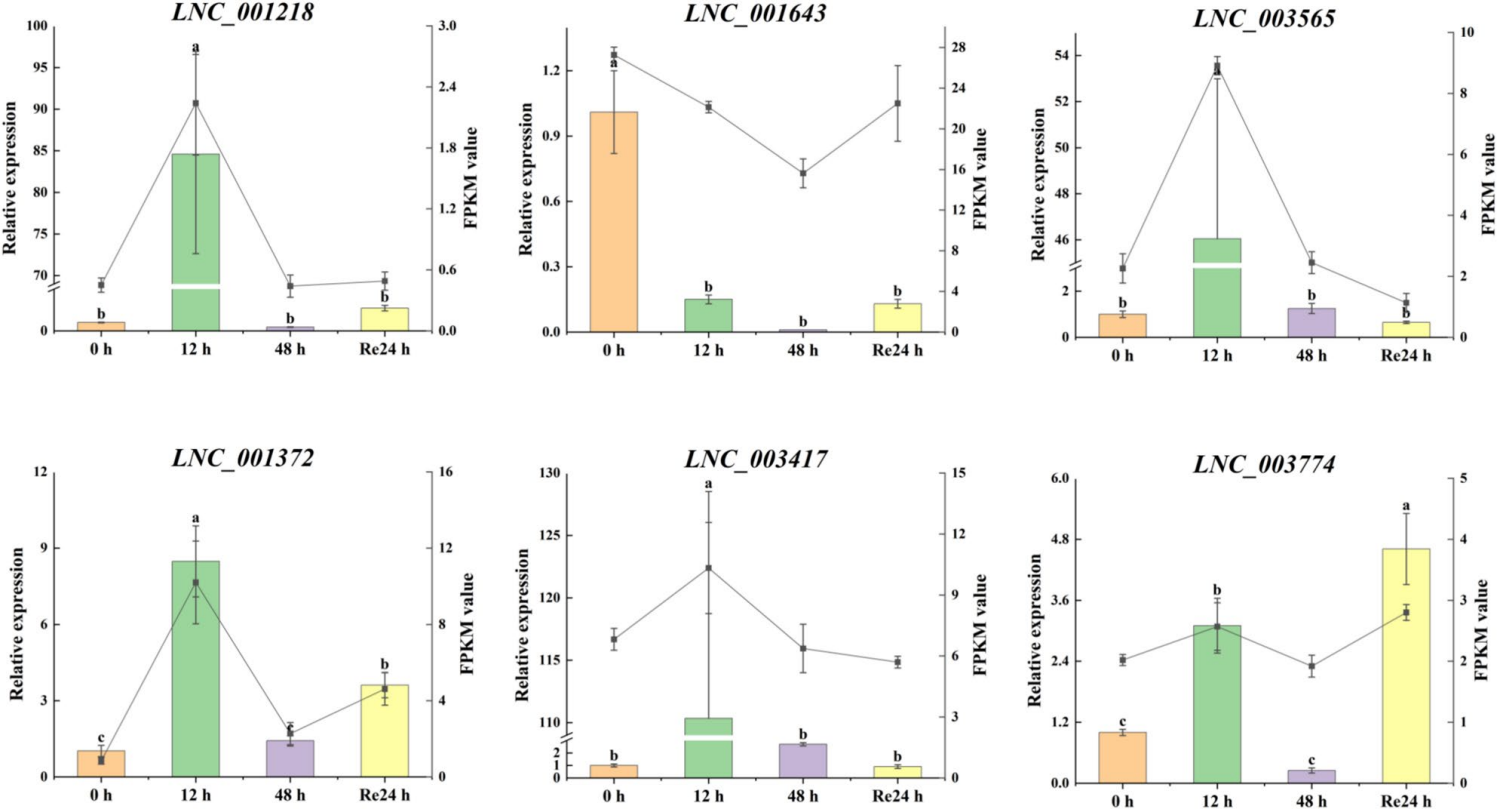
Selected lncRNAs were validated by RT-qPCR

To validate the RNA-seq data, the relative expression levels of six lncRNAs (Fig. 7) and 24 mRNAs (Supplementary Fig. 4) were determined by RT-qPCR. Although the selected lncRNAs and mRNAs exhibited different expression changes, the expression trends across the four treatments were consistent with the sequencing data, indicating that the sequencing data were reliable.

## Discussion

This study used Longzi purple Qingke as the material. It systematically investigated the response mechanisms of Qingke during 0℃ low-temperature stress and recovery through physiological and biochemical indicator measurements combined with transcriptomics (mRNA and lncRNA) analysis. The results indicate that Qingke responds to low-temperature stress by dynamically adjusting physiological metabolism, activating the antioxidant system, and reshaping gene expression networks (especially pathways related to photosynthesis and energy metabolism). lncRNA may play a key role in this process by regulating target genes. The following discussion delves deeper into the results in conjunction with existing research.

### Physiological regulatory mechanisms of Qingke in response to low-temperature stress

Low-temperature stress induces the production of a large amount of reactive oxygen species (ROS) in plant cells, leading to membrane lipid peroxidation (MDA accumulation) and protein structural damage. Plants counteract low-temperature damage by activating antioxidant enzyme systems (SOD, POD, CAT, etc.) to scavenge ROS, which is an important strategy [13–15, 26, 27]. In this study (Fig. 1), SOD and POD activity in Qingke significantly increased after 12 h of low-temperature stress, while MDA content gradually increased with prolonged stress duration, indicating that Qingke rapidly initiates antioxidant defense mechanisms under short-term low temperatures to mitigate ROS-induced cellular damage; However, after 48 h of stress, enzyme activity decreased and MDA accumulation slowed, possibly because prolonged low temperatures exceeded the regulatory threshold of the antioxidant system, prompting cells to shift toward maintaining homeostasis by accumulating osmotic regulatory substances (such as soluble proteins) [12]. After 24 h of recovery, SOD activity further decreased. In contrast, MDA content declined, suggesting that Qingke gradually repairs membrane damage at normal temperature, consistent with the phased “antioxidant-repair” response characteristic of plants after low-temperature stress [15].

The accumulation of soluble proteins and soluble sugars is another important physiological mechanism by which plants respond to low temperatures. It achieves cold resistance by increasing cell osmotic pressure, stabilizing biological membrane structures, and protecting enzyme activity [12]. In this study, the content of soluble proteins continued to increase under prolonged low-temperature stress. It remained higher than in the control group even after recovery, indicating that Qingke enhances stress resistance by continuously synthesizing protein-like substances. The dynamic changes in soluble sugars may be related to adjustments in energy metabolism, with short-term accumulation providing energy to cells and long-term accumulation potentially contributing to osmotic regulation. The synergistic changes in these physiological indicators reveal that Qingke adapts to low-temperature stress through a multi-level strategy involving “antioxidant defense-osmotic regulation-damage repair.”

### Transcriptome reveals core pathways and genes involved in low-temperature response

Transcriptome analysis revealed that low-temperature stress induced a large number of differentially expressed mRNAs. The response patterns at different treatment stages showed significant differences(Fig. 2): the highest number of differentially expressed genes (1,697) was observed under short-term (12 h) stress, with downregulated genes accounting for 80.02% of the total, which may be related to the rapid inhibition of non-essential metabolism by cells to conserve energy. Under long-term (48 h) stress, the number of differentially expressed genes decreased (from 863 to 863), and the proportion of up-regulated genes increased (from 35.8% to 35.8%), suggesting that plants gradually activate adaptive metabolic pathways [10, 16, 17].

GO enrichment analysis indicated that differentially expressed genes were mainly involved in biological processes (Supplementary Fig. 2) such as “cellular processes,” “metabolic processes,” and “stress responses,” as well as molecular functions such as “catalytic activity” and “binding function.” This is consistent with the need for cells to remodel metabolic networks and activate signal transduction under low-temperature conditions [28]. For example, enrichment for genes related to “catalytic activity” suggests that Qingke responds to stress by adjusting the efficiency of enzymatic reactions (e.g., antioxidant and metabolic enzymes), which is consistent with changes in enzyme activity observed in physiological indicators.

KEGG pathway analysis further revealed specific molecular mechanisms (Fig. 3): short-term stress (0 h vs. 12 h): plant hormone signaling pathways were significantly enriched, indicating that early low temperatures may initiate stress responses through hormone-mediated signaling networks such as ABA and BR [29]; the enrichment of plant-pathogen interaction pathways suggests that low temperatures may enhance plant sensitivity to pathogen invasion or that there may be common defensive signal crosstalk between the two [29]. Long-term stress (0 h vs. 48 h): Enrichment of the ribosomal pathway (translation process) and phenylpropanoid biosynthesis pathway suggests that Qingke enhances cold tolerance by increasing protein synthesis (repairing damaged proteins) and secondary metabolism (e.g., accumulation of phenolic compounds), similar to the key roles of ribosomal function and phenylpropanoid metabolism in rice cold responses [28].

Recovery phase (48 h vs. Re24h): Carbon metabolism and fatty acid degradation pathways were enriched, indicating that Qingke provides energy by breaking down stored substances (such as fatty acids) while reshaping carbon metabolism to restore the balance between photosynthesis and respiration, reflecting a strategic shift from “defense” to “growth recovery.”

Qingke’s response to cryostress is a complex regulatory network involving multipathway synergy, with hormone signaling, photosynthesis, energy metabolism, and phenylpropane biosynthesis pathways playing key roles.

Hormone signaling plays an early priming role in the barley cold response. Transcriptome analysis showed that the plant hormone signal transduction pathway was the most significantly up-regulated enrichment pathway after 3 days and 7 days of low-temperature treatment [30]. This finding suggests that hormone signaling may play a central regulatory role in the cellular reprogramming process induced by barley’s low temperature. In particular, the jasmonic acid signaling pathway became the dominant pathway after 12 h of stress, indicating that it rapidly activates downstream defense responses as an early signaling molecule. In addition, the strong expression of the calcium-dependent protein kinase (CDPK) gene under low-temperature stress further confirms the importance of calcium-mediated signal transduction in cold resistance [31].

Photosynthesis is one of the physiological processes most affected by low-temperature stress in barley. Low temperatures significantly inhibit plants’ ability to assimilate carbon through photosynthesis. The target genes of all lncRNAs that are differently expressed in response to low-temperature stress and are related to photosynthesis show increased expression levels; these include genes such as PSbA and PSbB in photosystem II and photosystem I, as well as PetB, PetD, PetA, and others (Fig. 6). After being exposed to low temperatures for 48 h, the net photosynthetic rate can decrease by 73.24%, and stomatal conductance can be reduced by 83.87% [32]. At the same time, the chlorophyll content decreased by 36.10%, and the structure of thylakoids and other photosynthetic membranes changed [30]. However, the reversibility of the photosynthetic system is also a key indicator of recovery – photosynthetic parameters can be significantly restored after the temperature rises [32]. In addition, differential lncRNAs (such as LNC_003210) have been found to target photosynthesis-related genes, revealing new post-transcriptional mechanisms of photosynthetic regulation.

Reprogramming of energy metabolism is an important strategy for barley to adapt to low temperatures. Under low-temperature stress, carbohydrate metabolism was significantly enriched, but metabolic flow was redirected [30]. It was found that starch synthesis was inhibited during cold nights, and malic acid replaced starch as the second contributor to meeting the nighttime carbon demand, accounting for 24–28% of the total carbon consumption [33]. The activation of carbon metabolism and fatty acid degradation pathways after 24 h of recovery reflects the rapid recovery mechanism of energy metabolism.

In barley’s cold resistance, the phenylpropane biosynthesis pathway is primarily involved in secondary metabolite accumulation and membrane lipid remodeling. Metabolomics analysis has revealed that under low-temperature stress, the phenylpropane metabolic pathway is significantly enriched, and phenolic compounds such as sinapoyl putrescine show marked increases in their levels [32]. At the same time, membrane lipidomics studies revealed the key role of membrane lipid modification: phosphatidic acid (PA), lysophosphatidic acid (LPA), and monogalactodiacylglycerol (MGDG) were significantly up-regulated under freezing stress, while the sensitive varieties decreased [34]. The expression of diacylglycerol acyltransferase 1 (DGAT1) was significantly increased in frost-tolerant cultivars, and SENSITIVE TO FREEZING 2 (SFR2) reached its highest level after 48 h of cold treatment, which together maintained membrane fluidity and integrity at low temperatures [34].

Hormone signals initiate stress response, photosynthesis provides the energy basis but is inhibited by low temperature, energy metabolism maintains the balance between supply and demand through carbon flow redistribution, and phenylpropane metabolism enhances structural stability through secondary product and membrane lipid remodeling. The enhancement of ribosomal (protein synthesis) and phenylpropane metabolism after 48 h of stress complements the activation of carbon metabolism and fatty acid degradation at Re24h, which together constitute a complete regulatory framework that enables barley to cope with low-temperature stress and restore growth.

### The regulatory role of lncRNA in the low-temperature response of Qingke

Initially, lncRNAs were considered a form of transcriptional noise, a class of non-coding RNAs that encode only short peptides. Their expression levels in organisms are lower than those of mRNAs. However, subsequent studies have revealed that lncRNAs are widely involved in plant growth and development, signal transduction, and stress responses [35]. As important epigenetic regulatory factors, lncRNAs participate in plant stress responses by targeting mRNAs [29]. This study found that the differential expression patterns of Qingke lncRNAs under low-temperature stress were similar to those of mRNAs (e.g., 85.9% of lncRNAs were upregulated at 48 h of stress). The functional enrichment of their target genes revealed the core regulatory direction of lncRNAs: photosynthesis and energy metabolism. Differentially expressed lncRNA target genes were significantly enriched in photosynthesis (photosystem I/II, ATP synthase) and oxidative phosphorylation pathways at all treatment stages, with most target genes showing upregulation (e.g., PSbA, PSbB, PetA). This suggests that lncRNAs may maintain energy supply under low temperatures by enhancing the expression of photosynthesis-related genes—a finding consistent with Qingke’s long-term adaptation to high-altitude low-temperature environments, where it optimizes energy metabolism efficiency to withstand stress. For example, the upregulation of ATPase-related lncRNAs (such as LNC_002865) may alleviate ATP synthesis inhibition caused by low temperatures by activating target gene expression [13]. Redox balance: The enrichment of target genes in the oxidative phosphorylation pathway further supports the involvement of lncRNAs in ROS clearance and cellular homeostasis through the regulation of energy metabolism-related genes (such as respiratory chain complexes) [15]. Additionally, the 11 lncRNAs and their 75 target genes (enriched in the chloroplast and ribosome pathways) that were differentially expressed in multiple treatment groups may be core regulatory factors in Qingke’s response to low temperatures, providing key candidates for subsequent functional validation. The study further conducted an in-depth analysis using LNC_003210 as a representative case, revealing that its target genes are all associated with chloroplast function. Sequence alignment analysis uncovered its potential mechanism of action: it exhibits sequence homology with some target genes, suggesting it may function via a ceRNA (competitive endogenous RNA) mechanism; conversely, the absence of sequence repeats with other target genes implies the existence of alternative trans-regulatory mechanisms. This study not only provides valuable candidate lncRNA resources to elucidate the cold-tolerance mechanism in Qingke but also, through enrichment analysis and case studies, offers strong evidence supporting the central role of LNC_003210 in light response and regulation of chloroplast function. This lays a solid foundation for subsequent functional validation experiments.

### The role of the integrated regulatory network of lncRNA and mRNA in cold resistance of Qingke

During barley’s response to low-temperature stress and the restoration of growth, lncRNA and mRNA form a complex, synergistic regulatory network that jointly mediates post-transcriptional gene expression. Comprehensive transcriptome analysis revealed significant co-expression between differentially expressed lncRNAs and mRNAs, both of which were jointly involved in regulating multiple key biological pathways.

The targeted regulation of photosynthesis by lncRNAs is an important regulatory mode found in this study. Differential lncRNAs LNC_003210 were identified as potential targeted regulators of photosynthesis-related genes, suggesting that lncRNAs may affect the functional state of the photosynthetic system through cis- or trans-mechanisms of action. Studies have shown that lncRNAs function as “biological regulators” in plant cold stress responses, targeting a variety of stress-response mRNAs and regulatory genes at the transcriptional, post-transcriptional, and epigenetic levels [36]. Under low temperature stress, the expression of photosynthesis-related mRNAs changed significantly, and the differential expression of lncRNAs may serve as upstream regulatory signals, mediating the rapid response of photosynthetic genes. The existence of this lncRNA-mRNA regulatory module provides a post-transcriptional explanation for the reversible inhibition and recovery of Qingke’s photosynthetic system under low temperature stress.

LncRNAs are involved in the synergistic regulation of stress signaling pathways. In hormone signaling and pathogen defense pathways, not only were core differential mRNAs significantly enriched, but also multiple differential lncRNAs were found to co-express with the coding genes of these pathways. Recent studies have found that lncRNAs can mediate interactions between the calcium signaling system and the transcriptional cascade, while coordinating hormone signaling networks, including abscisic acid, jasmonic acid, ethylene, salicylic acid, and brassinolactone [37]. The expression pattern of jasmonic acid signaling pathway-related genes became the dominant pathway after 12 h of stress, which was highly correlated with the dynamic changes of specific lncRNAs, suggesting that lncRNAs may participate in the cascade amplification of stress signals by affecting the expression of signal transduction factors or transcription factors.

The construction of the temporal regulatory network revealed the dynamic synergy of lncRNA and mRNA in the stress process. After 48 h of stress, ribosome (protein synthesis) and phenylpropane metabolism (secondary metabolites) pathways were enhanced. They may differentially express lncRNAs at this stage to regulate protein translation efficiency and secondary product accumulation by modulating the expression of ribosomal protein or key enzyme genes involved in phenylpropane metabolism. After 24 h of recovery, the carbon metabolism and fatty acid degradation pathways were activated, and the corresponding lncRNA-mRNA co-expression modules were also transformed, reflecting the time-series specificity of the regulatory network.

LncRNA and mRNA form a multi-level regulatory network in barley cold resistance: lncRNA directly targets photosynthetic genes to regulate photosynthetic efficiency, responds to hormone signals in synergy with stress signaling pathway mRNA, dynamically regulates protein synthesis and secondary metabolism in the timing process, and may indirectly affect the expression of energy metabolism-related genes through the ceRNA mechanism. This comprehensive regulatory model of lncRNA-mRNA provides a regulatory basis for barley to adapt to low-temperature stress and restore growth at the post-transcriptional level, and also offers a new perspective for in-depth analysis of the molecular mechanism of plant cold resistance.

### Synergy between physiological and molecular mechanisms

In this study, physiological indicators and transcriptomic data showed a high degree of consistency: for example, the accumulation of soluble substances was consistent with the upregulation trend of genes involved in sugar metabolism and amino acid metabolism pathways; and the enrichment of lncRNA target genes in the photosynthesis pathway provided a molecular explanation for the maintenance of energy metabolism at the physiological level. This “physiological-molecular” synergistic response reflects Qingke’s systemic adaptive strategy to cold stress: in the short term, it primarily involves rapid activation of defense systems (antioxidant, hormone signaling); in the long term, it consolidates cold tolerance through metabolic restructuring (photosynthetic optimization, secondary metabolism); and during the recovery phase, it achieves functional repair through energy metabolism reconstruction.

In summary, this study demonstrates that Qingke responds to low-temperature stress by dynamically adjusting its antioxidant system, osmotic substance accumulation, and gene expression network (particularly pathways related to photosynthesis and energy metabolism), with lncRNA playing a significant regulatory role. The central role of LNC_003210 in light response and chloroplast function regulation during cold stress is highlighted. The results provide a theoretical basis and candidate gene resources for in-depth analysis of Qingke’s cold-tolerance mechanism and for breeding cold-tolerant varieties.

## Materials and methods

### Experimental design and treatment conditions

The material used in this study, Longzi purple Qingke, is a purple-grain Qingke variety cultivated by farmers in Longzi County, Shannan Prefecture, Xizang Autonomous Region. Seeds were selected for their plumpness and freedom from pests. They were disinfected with 10% H₂O₂ for 10 min, rinsed with sterile water 2–3 times, and placed in a petri dish lined with two layers of filter paper for germination. Once the seeds sprouted, they were transplanted into pots (10 cm × 12 cm) filled with a 1:1 mixture of seedling medium and soil and placed in an artificial climate chamber for routine management (14 h of light followed by 10 h of darkness, with temperatures maintained at 24℃during the day and 18℃at night). The plants were cultivated until they reached the three-leaf stage, after which they were subjected to low-temperature treatment (0 °C, with 14 h of light followed by 10 h of darkness). Leaves were collected at various time points: 0 h (control check, CK), 12 h, 48 h, and 24 h after recovery at 24℃following 48 h of low-temperature stress (Recovery 24 h). The experiment was set up with 3 replications. The leaves were then quickly frozen in liquid nitrogen and stored in an ultra-low-temperature freezer for subsequent use (specifically for LncRNA analysis).

### Measurement of physiological and biochemical indicators

Enzyme extraction: Remove the sample from the − 80 °C freezer, accurately weigh 0.5 g into a clean mortar, add 5 mL phosphate buffer, and grind in an ice bath until homogenized. Transfer the mixture to a 10 mL centrifuge tube, centrifuge at low speed for 10 min, transfer the supernatant to a new centrifuge tube, and store at 4 °C for later use [27]. The determination of superoxide dismutase (SOD) was performed according to the method described by Giannopolitis et al. [38], the determination of peroxidase (POD) was performed according to the method described by Maehly et al. [39], the determination of CAT was performed according to the method described by Maehly et al. [39], and the determination of MDA was performed using the Hodges method [40]. Relative Electrical Conductivity (REC) was measured according to the method of Shi [41], Proline was measured according to the method of Nguyen et al. [42], and soluble protein was measured using Noble’s method [43]. APX activity was measured according to the method of Saruyama and Tanida [44]. PAL activity was measured according to the method of Ferrarese [45]. Data were analyzed by one-way analysis of variance (ANOVA) followed by Duncan’s multiple range test using SPSS 20.0 (IBM, USA). Statistical graphs were generated using GraphPad Prism 10 (GraphPad Software, USA). A *p*-value < 0.05 was considered statistically significant.

### RNA extraction, library construction, and sequencing

Total RNA was extracted from 12 samples. Library preparation and lncRNA sequencing were performed by Metware Biotechnology Co., Ltd. (Wuhan, China). RNA Kit removes rRNA. The fragmentation buffer was then added to break the RNA into short fragments of 250–300 bp, using the short fragment RNA as a template, and the single-stranded cDNA was synthesized using six-base random primers, followed by AMPure XP beads to purify the double-stranded cDNA. The purified double-stranded cDNA is then end-repaired, A-tailed, and attached to the sequencing adapter, followed by fragment size selection with AMPure XP beads. The USER enzyme then degraded the second strand of cDNA containing U, and finally, PCR enrichment was performed to obtain a strand-specific cDNA library. The data generated after sequencing is first filtered to remove reads containing adapters. Reads with N content exceeding 1% are filtered out, as well as reads that form paired-end pairs. When the number of low-quality bases (≤ 20) in a single-end sequencing read exceeds 50% of the read length, that read and the read that forms a paired-end pair with it are filtered out. The result is clean reads. The clean reads are aligned with the reference genome (Genome assembly Hulless_Barley_ass. V2, GCA_004114815.1), and the mapping rate is calculated. We stored these data in the NCBI database (https://www.ncbi.nlm.nih.gov/Traces/index.html, Accession Number: PRJNA1377543).

### Transcript reconstruction and screening

Transcript assembly was performed using the StringTie software [46]. The assembled GTF files were merged using cuffmerge [47] to remove redundancy, followed by lncRNA identification and screening. Based on the above results, cuffcompare was used to compare the reference genome annotation file, screen for known lncRNAs and known mRNAs in the reference genome annotation file and predict new transcripts. Finally, the screened novel transcripts were subjected to transcript length screening and coding potential prediction, yielding the final Novel lncRNA and Novel mRNA.

Based on the transcriptome splicing results, according to the structural characteristics of lncRNAs and the functional characteristics of non-coding proteins, we set a series of strict screening conditions to screen the spliced transcripts, and the final screened lncRNAs were used as the final novel lncRNAs for subsequent analysis, the specific screening conditions are as follows:

1. Transcripts with a length greater than or equal to 200 bp.
2. Use cuffcompare to filter transcripts with overlapping regions of mRNA and other non-coding RNAs (rRNA, tRNA, etc.) in the known database, if there is an annotation of lncRNA in the database, we will include the lncRNA in the database that overlaps with the exon region of the spliced transcript as known lncRNA in the subsequent analysis;
3. For novel lncRNAs, according to their lack of coding potential as a condition for judging whether they are lncRNAs, three coding potential prediction software, CNCI [46], CPC2 [48], and PLEK [49] were used to predict the coding potential of transcripts, and the prediction results were intersected.

### lncRNA target gene prediction

Set the threshold for cis-target gene screening based on positional relationships to 100 kb upstream and downstream of lncRNA. Subsequently, functional enrichment analysis was performed on the mRNA target genes predicted based on the position of lncRNA. The Pearson correlation coefficient method was used to analyze the correlation between lncRNAs and mRNAs in samples to screen trans-target genes. mRNA with an absolute correlation value greater than 0.95 and *P*-value < 0.01 was selected as its target gene for functional enrichment analysis to predict the primary function of lncRNA.

### Differential expression analysis and enrichment analysis of lncRNA and mRNA

The expression levels of transcripts were calculated using the StringTie software, which provided the number of reads mapped to each transcript (including lncRNA and mRNA) in each sample. These counts were then converted to FPKM (Fragments Per Kilobase of transcript per Million mapped reads) [47]. Standardize the raw read counts, then use a statistical model to calculate the *P*-value for hypothesis testing, and finally perform multiple hypothesis testing correction to obtain the FDR value. The significance analysis of transcript expression levels was conducted using DESeq2 [50], applying the following screening criteria: | log2FoldChange | ≥ 1 and *P*-value < 0.05.

### RT-qPCR analysis

To further validate the accuracy of the transcriptomic data, 6 lncRNAs and 25 mRNA genes were selected for RT-qPCR analysis. The primer sequences used are listed in Supplementary Table 1. Using the transcriptomic sequencing samples, cDNA was first synthesized using the SweScript I First Strand cDNA Synthesis Kit from Wuhan Servicebio Technology Co., Ltd. (Wuhan, China) via reverse transcription. The procedure followed the kit instructions with minor modifications; Second, the SYBR Green Premix Pro Taq HS qPCR Kit Accurate Biotechnology (HUNAN) Co., Ltd. (ChangSha, China) was used for amplification experiments (BIO-RAD T100 Thermal Cycler). The *tuba* reference gene primers, which were identified as stable internal controls through screening by Cai [51] were employed in this study. The primer sequences were:

- F: CCATCAAGACCAAGCGCACTA
- R: CATACCCTCACCCACATACCA

Finally, the gene abundance was calculated using the 2^−ΔΔCT^ method [52], with all experimental treatments repeated three times.

## Conclusion

Qingke responds to low-temperature stress through physiological mechanisms such as activating antioxidant defense, osmoregulation, and maintaining membrane stability, and sustained stress responses persist during the recovery period. The differential gene and lncRNA expression induced by low-temperature stress exhibits time-specific dynamics, with core regulatory networks involving hormone signaling, photosynthesis, energy metabolism, and phenylpropanoid biosynthesis pathways. LNC_003210 may play a key role in Qingke cold adaptation by regulating genes related to photosynthesis metabolism.

## Supplementary Information

Supplementary Material 1

Supplementary Material 2

Supplementary Material 3

Supplementary Material 4

Supplementary Material 5

Supplementary Material 6

Supplementary Material 7

## Data Availability

The lncRNA sequencing data generated in this study have been deposited in the NCBI Sequence Read Archive (SRA) under the BioProject accession number PRJNA1377543. The data are publicly available in the NCBI database.

## References

[1] He Q, Wang XM, He L, Yang L, Wang S, Bi Y. Alternative respiration pathway is involved in the response of highland barley to salt stress. Plant Cell Rep. 2019;38:295–309. 10.1007/s00299-018-2366-6. 30542981 10.1007/s00299-018-2366-6

[2] Bao L, Bao GZ, Zhang X, Qu Y, Guo J, Pan X. Short-term effects of combined freeze–thaw and saline–alkali stresses on the physiological response in highland barley (*Hordeum vulgare*). Funct Plant Biol. 2022;49:970–9. 10.1071/FP22097. 35892141 10.1071/FP22097

[3] He Y, Wang AX, Chen ZY, Nie M, Xi H, Gong X, et al. Effects of egg powder on the structure of highland barley dough and the quality of highland barley bread. Int J Biol Macromol. 2023;240:124376. 10.1016/j.ijbiomac.2023.124376. 37059285 10.1016/j.ijbiomac.2023.124376

[4] He Y, Wang AX, Qin WY, Chen ZY, Xi H, Nie M, et al. Effects of semidry milling on the properties of highland barley flour and the quality of highland barley bread. J Sci Food Agric. 2023;103:5077–86. 10.1002/jsfa.12586. 36990966 10.1002/jsfa.12586

[5] Obadi M, Qi YJ, Xu B. Highland barley starch (qingke): structures, properties, modifications, and applications. Int J Biol Macromol. 2021;185:725–38. 10.1016/j.ijbiomac.2021.06.204. 34224757 10.1016/j.ijbiomac.2021.06.204

[6] Chang YX, Zhang JT, Bao GZ, Yan B, Qu Y, Zhang M, et al. Physiological responses of highland barley seedlings to NaCl, drought, and freeze-thaw stress. J Plant Growth Regul. 2021;40:154–61. 10.1007/s00344-020-10085-5.

[7] R Pearce. Plant freezing and damage. Ann Bot. 2001;87:417–24. 10.1006/anbo.2000.1352.

[8] Ding YL, Shi YT, Yang SH. Advances and challenges in uncovering cold tolerance regulatory mechanisms in plants. New Phytol. 2019;222:1690–704. 10.1111/nph.15696. 30664232 10.1111/nph.15696

[9] Jini D, Joseph B. Physiological mechanism of salicylic acid for alleviation of salt stress in rice. Rice Sci. 2017;24:97–108. 10.1016/j.rsci.2016.07.007.

[10] Saharan BS, Brar B, Duhan JS, Kumar R, Marwaha S, Rajput VD, et al. Molecular and physiological mechanisms to mitigate abiotic stress conditions in plants. Life. 2022;12:1634. 10.3390/life12101634. 36295069 10.3390/life12101634 PMC9605384

[11] Zhang Q, Li DM, Wang Q, Song X, Wang Y, Yang X, et al. Exogenous salicylic acid improves chilling tolerance in maize seedlings by improving plant growth and physiological characteristics. Agronomy. 2021;11:1341. 10.3390/agronomy11071341.

[12] Allen DJ, Ort DR. Impacts of chilling temperatures on photosynthesis in warm-climate plants. Trends Plant Sci. 2001;6:36–42. 10.1016/S1360-1385(00)01808-2. 11164376 10.1016/s1360-1385(00)01808-2

[13] Airaki M, Leterrier M, Mateos RM, Valderrama R, Chaki M, Barroso JB, et al. Metabolism of reactive oxygen species and reactive nitrogen species in pepper (*Capsicum annuum* L.) plants under low temperature stress. Plant Cell Environ. 2012;35:281–95. 10.1111/j.1365-3040.2011.02310.x. 21414013 10.1111/j.1365-3040.2011.02310.x

[14] Lukatkin AS. Contribution of oxidative stress to the development of cold-induced damage to leaves of chilling-sensitive plants: 1. Reactive oxygen species formation during plant chilling. Russ J Plant Physiol. 2002;49:622–7. 10.1023/A:1020232700648.

[15] Chi YX, Yang L, Zhao CJ, Muhammad I, Bo Zhou X, De Zhu H. Effects of soaking seeds in exogenous vitamins on active oxygen metabolism and seedling growth under low-temperature stress. Saudi J Biol Sci. 2021;28:3254–61. 10.1016/j.sjbs.2021.02.065. 34121863 10.1016/j.sjbs.2021.02.065 PMC8176085

[16] Guan Y, Hwarari D, Korboe HM, Ahmad B, Cao Y, Movahedi A, et al. Low temperature stress-induced perception and molecular signaling pathways in plants. Environ Exp Bot. 2023;207:105190. 10.1016/j.envexpbot.2022.105190.

[17] Ritonga FN, Chen S. Physiological and molecular mechanism involved in cold stress tolerance in plants. Plants. 2020;9:560. 10.3390/plants9050560. 32353940 10.3390/plants9050560 PMC7284489

[18] Jin J, Liu J, Wang H, Wong L, Chua N-H, PLncDB. Plant long non-coding RNA database. Bioinformatics. 2013;29:1068–71. 10.1093/bioinformatics/btt107. 23476021 10.1093/bioinformatics/btt107 PMC3624813

[19] Ariel F, Jegu T, Latrasse D, Romero-Barrios N, Christ A, Benhamed M, et al. Noncoding transcription by alternative RNA polymerases dynamically regulates an auxin-driven chromatin loop. Mol Cell. 2014;55:383–96. 10.1016/j.molcel.2014.06.011. 25018019 10.1016/j.molcel.2014.06.011

[20] Tian M, Ci D, Song Y, Zhang D. Transcriptional regulation of chilling stress responsive long noncoding RNAs in populus simonii. Trees. 2019;33:733–49. 10.1007/s00468-019-01812-x.

[21] Wang P, Dai L, Ai J, Wang Y, Ren F. Identification and functional prediction of cold-related long non-coding RNA (lncRNA) in grapevine. Sci Rep. 2019;9:6638. 10.1038/s41598-019-43269-5. 31036931 10.1038/s41598-019-43269-5 PMC6488645

[22] Baruah PM, Agarwala N, Bordoloi KS, Regon P, Tanti B. Long non-coding RNAs responsive to temperature stress conditions in tea plants. J Plant Growth Regul. 2025;44:1728–52. 10.1007/s00344-024-11444-2.

[23] Zhu M, Dong Q, Bing J, Songbuerbatu, Zheng L, Dorjee T, et al. Combined lncRNA and mRNA expression profiles identified the lncRNA–miRNA–mRNA modules regulating the cold stress response in ammopiptanthus nanus. Int J Mol Sci. 2023;24:6502. 10.3390/ijms24076502. 37047474 10.3390/ijms24076502 PMC10095008

[24] Porras-Dominguez J, Lothier J, Limami AM, Tcherkez G. d-amino acids metabolism reflects the evolutionary origin of higher plants and their adaptation to the environment. Plant Cell Environ. 2024;47:1503–12. 10.1111/pce.14826. 38251436 10.1111/pce.14826

[25] Kolukisaoglu Ü. d-amino acids in plants: sources, metabolism, and functions. Int J Mol Sci. 2020;21. 10.3390/ijms21155421. 10.3390/ijms21155421 PMC7432710 32751447

[26] Lee HJ, Lee JH, Wi S, Jang Y, An S, Choi CK, et al. Exogenously applied glutamic acid confers improved yield through increased photosynthesis efficiency and antioxidant defense system under chilling stress condition in *solanum lycopersicum* L. cv. Dotaerang dia. Sci Hortic. 2021;277:109817. 10.1016/j.scienta.2020.109817.

[27] Sin’kevich MS, Selivanov AA, Antipina OV, Kropocheva EV, Alieva GP, Suvorova TA, et al. Activities of antioxidant enzymes of arabidopsis thaliana plants during cold hardening to hypothermia. Russ J Plant Physiol. 2016;63:749–53. 10.1134/S1021443716060108.

[28] Li Z, Khan MU, Letuma P, Xie Y, Zhan W, Wang W, et al. Transcriptome analysis of the responses of rice leaves to chilling and subsequent recovery. Int J Mol Sci. 2022;23:10739. 10.3390/ijms231810739. 36142652 10.3390/ijms231810739 PMC9502032

[29] Zhao W, Xiao W, Sun J, Chen M, Ma M, Cao Y, et al. An Integration of MicroRNA and Transcriptome Sequencing Analysis Reveal Regulatory Roles of miRNAs in Response to Chilling Stress in Wild Rice. Plants. 2022;11:977. 10.3390/plants11070977. 35406957 10.3390/plants11070977 PMC9002458

[30] Transcriptomic analysis reveals the effect of cold stress on the totipotency of barley microspores. Acta Agric Shanghai. 2024;40:7–16. 10.15955/j.issn1000-3924.2024.01.02.

[31] Zhao L, Cassan-Wang H, Zhao Y, Bao Y, Hou Y, Liu Y, et al. Calcium-dependent protein kinase PpCDPK29-mediated Ca2+-ROS signal and PpHSFA2a phosphorylation regulate postharvest chilling tolerance of peach fruit. Plant Biotechnol J. 2025;23:1938–53. 10.1111/pbi.70024. 10.1111/pbi.70024 PMC12120869 40014693

[32] Yu M, Luobu Z, Zhuoga D, Wei X, Tang Y. Physiological and broadly targeted metabolomic analyses of barley (hordeum vulgare L.) in response to low-temperature stress. BMC Genomics. 2025;26:618. 10.1186/s12864-025-11516-x. 40597568 10.1186/s12864-025-11516-x PMC12210896

[33] Barros KA, Esteves-Ferreira AA, Inaba M, Meally H, Finnan J, Barth S, et al. Diurnal patterns of growth and transient reserves of sink and source tissues are affected by cold nights in barley. Plant Cell Environ. 2020;43:1404–20. 10.1111/pce.13735. 32012288 10.1111/pce.13735

[34] Zhao Y, Li S, Wu J, Liu H, Wang P, Xu L. Insights into membrane lipids modification in barley leaves as an adaptation mechanism to cold stress. Plant Growth Regul. 2024;103:369–88. 10.1007/s10725-023-01114-w.

[35] Traubenik S, Charon C, Blein T. From environmental responses to adaptation: the roles of plant lncRNAs. Plant Physiol. 2024;kiae034. 10.1093/plphys/kiae034. 10.1093/plphys/kiae034 38246143

[36] Jha UC, Nayyar H, Jha R, Khurshid M, Zhou M, Mantri N, et al. Long non-coding RNAs: emerging players regulating plant abiotic stress response and adaptation. BMC Plant Biol. 2020;20. 10.1186/s12870-020-02595-x. 10.1186/s12870-020-02595-x PMC7549229 33046001

[37] Wielogórska M, Rucińska A, Kloc Y, Boczkowska M. Long non-coding RNAs in the cold-stress response of horticultural plants: molecular mechanisms and potential applications. Int J Mol Sci. 2025;26. 10.3390/ijms262110464. 10.3390/ijms262110464 PMC12608027 41226504

[38] Giannopolitis CN, Ries SK. Superoxide dismutases: I. Occurrence in higher plants. Plant Physiol. 1977;59:309–14. 10.1104/pp.59.2.309. 16659839 10.1104/pp.59.2.309 PMC542387

[39] Maehly AC. The assay of catalases and peroxidases. Methods of Biochemical Analysis. John Wiley & Sons, Ltd; 1954. pp. 357–424. 10.1002/9780470110171.ch14. 10.1002/9780470110171.ch14 13193536

[40] Hodges DM, DeLong JM, Forney CF, Prange RK. Improving the thiobarbituric acid-reactive-substances assay for estimating lipid peroxidation in plant tissues containing anthocyanin and other interfering compounds. Planta. 1999;207:604–11. 10.1007/s004250050524. 10.1007/s00425-017-2699-3 28456836

[41] Shi YT, Tian SW, Hou LY, Huang X, Zhang X, Guo H, et al. Ethylene signaling negatively regulates freezing tolerance by repressing expression of *CBF* and type-a *ARR* genes in Arabidopsis. Plant Cell. 2012;24:2578–95. 10.1105/tpc.112.098640. 22706288 10.1105/tpc.112.098640 PMC3406918

[42] Nguyen HTT, Das Bhowmik S, Long H, Cheng Y, Mundree S, Hoang LTM. Rapid accumulation of proline enhances salinity tolerance in australian wild rice oryza australiensis domin. Plants. 2021;10:2044. 10.3390/plants10102044. 34685853 10.3390/plants10102044 PMC8540606

[43] Noble JE, Bailey MJA. Chapter 8 quantitation of protein. In: Burgess RR, Deutscher MP, editors. Methods in Enzymology. Academic; 2009. pp. 73–95. 10.1016/S0076-6879(09)63008-1. 10.1016/S0076-6879(09)63008-1 19892168

[44] Saruyama H, Tanida M. Effect of chilling on activated oxygen-scavenging enzymes in low temperature-sensitive and -tolerant cultivars of rice (*oryza sativa* L). Plant Sci. 1995;109:105–13. 10.1016/0168-9452(95)04156-O.

[45] Ferrarese MLL, Rodrigues JD, Ferrarese-Filho O. Phenylalanine ammonia-lyase activity in soybean roots extract measured by reverse-phase high performance liquid chromatography. Plant Biol. 2000;2:152–3. 10.1055/s-2000-9162.

[46] Sun L, Luo H, Bu D, Zhao G, Yu K, Zhang C, et al. Utilizing sequence intrinsic composition to classify protein-coding and long non-coding transcripts. Nucleic Acids Res. 2013;41:e166–166. 10.1093/nar/gkt646. 23892401 10.1093/nar/gkt646 PMC3783192

[47] Trapnell C, Williams BA, Pertea G, Mortazavi A, Kwan G, van Baren MJ, et al. Transcript assembly and quantification by RNA-seq reveals unannotated transcripts and isoform switching during cell differentiation. Nat Biotechnol. 2010;28:511–5. 10.1038/nbt.1621. 20436464 10.1038/nbt.1621 PMC3146043

[48] Kang Y-J, Yang D-C, Kong L, Hou M, Meng Y-Q, Wei L, et al. CPC2: a fast and accurate coding potential calculator based on sequence intrinsic features. Nucleic Acids Res. 2017;45:W12–6. 10.1093/nar/gkx428. 28521017 10.1093/nar/gkx428 PMC5793834

[49] Li A, Zhang J, Zhou Z. PLEK: a tool for predicting long non-coding RNAs and messenger RNAs based on an improved k-mer scheme. BMC Bioinformatics. 2014;15:311. 10.1186/1471-2105-15-311. 25239089 10.1186/1471-2105-15-311 PMC4177586

[50] Love MI, Huber W, Anders S. Moderated estimation of fold change and dispersion for RNA-seq data with DESeq2. Genome Biol. 2014;15:550. 10.1186/s13059-014-0550-8. 25516281 10.1186/s13059-014-0550-8 PMC4302049

[51] Cai Jing. Study on the effect of environmental temperature on rhythmic Expression of abiotic stress resistance genes in tibetan hulless barley. Master’s thesis. Xi'an: Northwest University; 2019. https://kns.cnki.net/kcms2/article/abstract?v=y_SiIdm5mqubIVvIvL9lvYWDciAVgF6u5J-5UQBcSUnbY-H_doz9TRqiUqgahq5AfHhBfxd-nPB9NrpsdljzMgu75EUSZllYmv-_Fr62T2TDlRMcQ07Eu32vlN5ZNNEMQ-OYLbNUnEdfaxgertr5trt9GP9MrwnUEaqPcyfzmcpgowXeP5FFeDGyOdCq3bMk&uniplatform=NZKPT&language=CHS.

[52] Livak KJ, Schmittgen TD. Analysis of relative gene expression data using real-time quantitative PCR and the 2 – ∆∆CT method. Methods. 2001;25:402–8. 10.1006/meth.2001.1262. 11846609 10.1006/meth.2001.1262

