# Supplementary figures and images for: Transcriptome analysis revealed lncRNA-mRNA modules responsive to low temperature stress in Qingke
Source: BMC Genomics. 2026 May 11;27:588. doi: 10.1186/s12864-026-12838-0 (PMC13335024; doi:10.1186/s12864-026-12838-0)

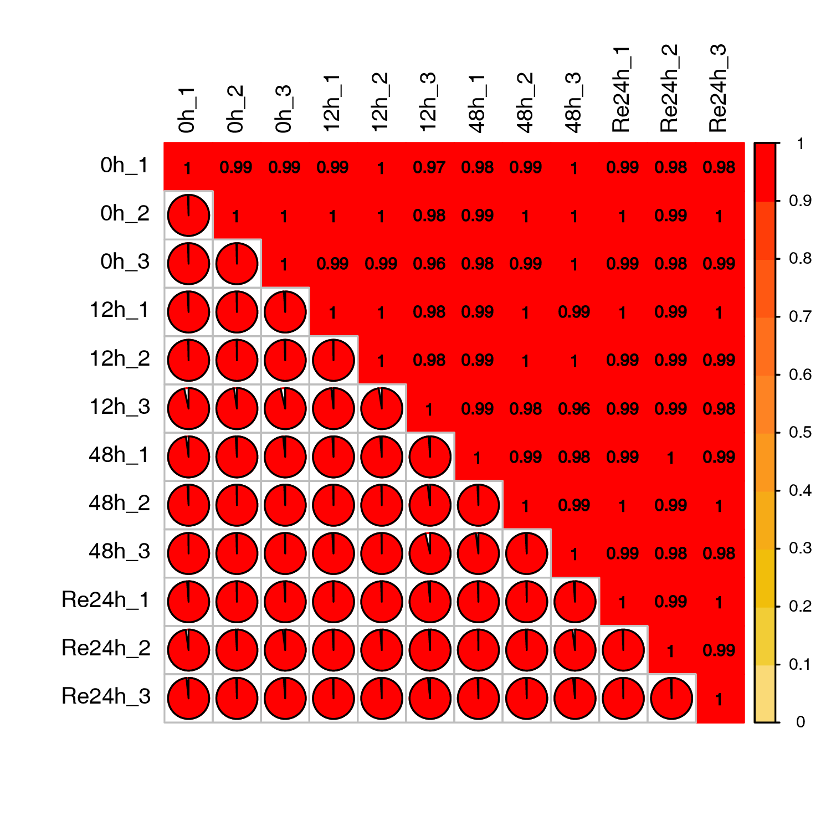

Supplement: Supplementary file 4 — Supplementary Material 4 [file 12864_2026_12838_MOESM4_ESM.png]

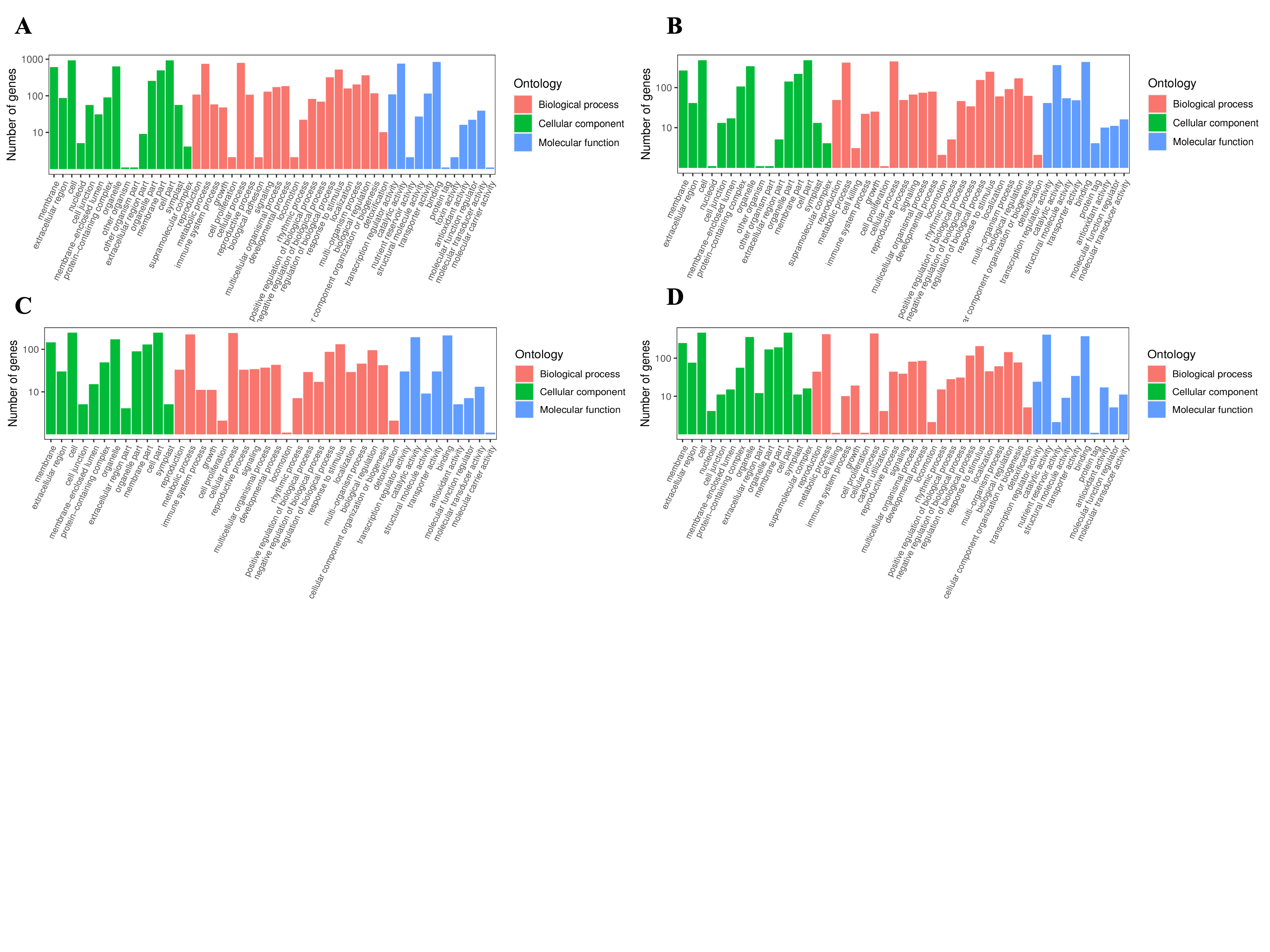

Supplement: Supplementary file 5 — Supplementary Material 5 [file 12864_2026_12838_MOESM5_ESM.tif]

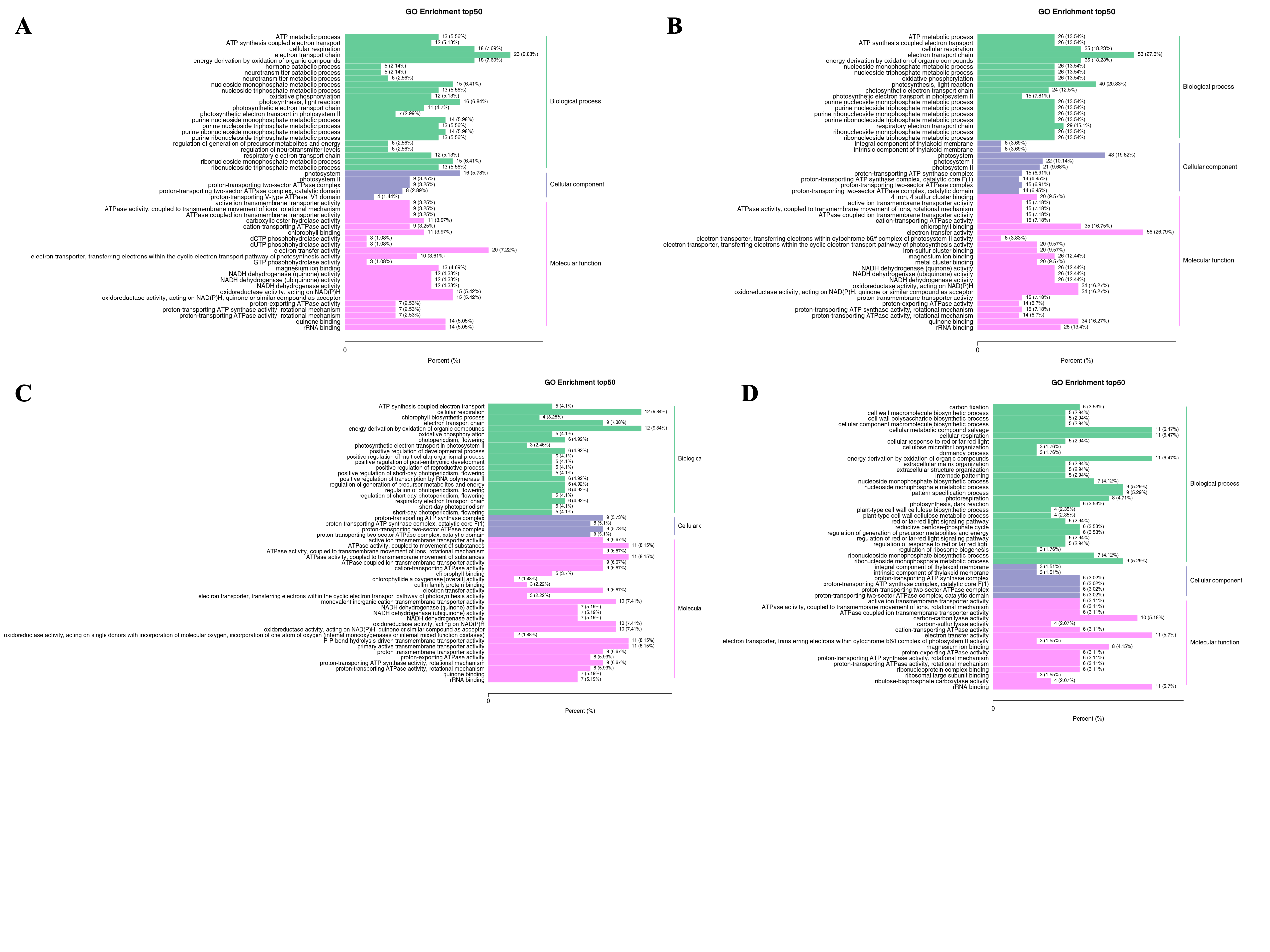

Supplement: Supplementary file 6 — Supplementary Material 6 [file 12864_2026_12838_MOESM6_ESM.tif]

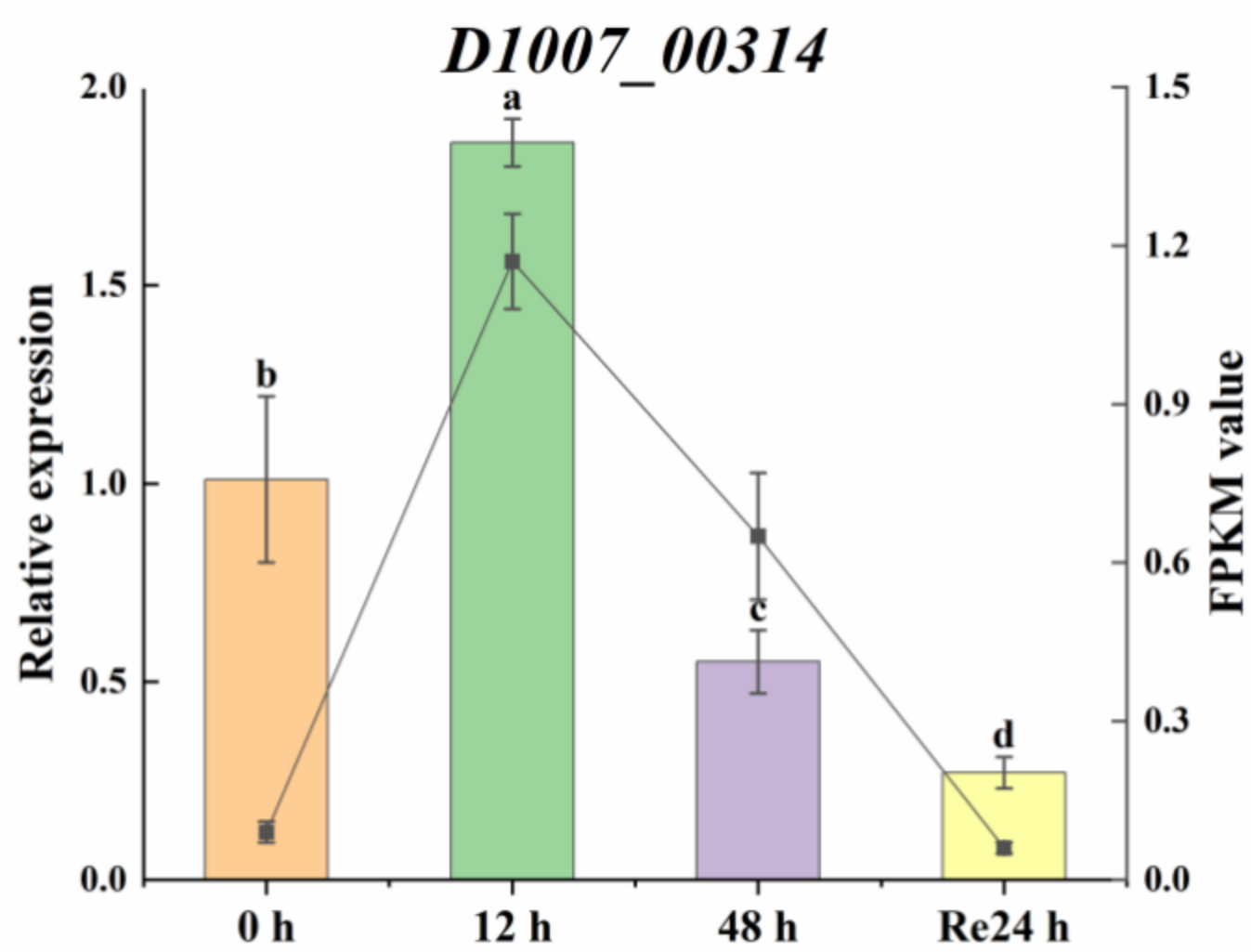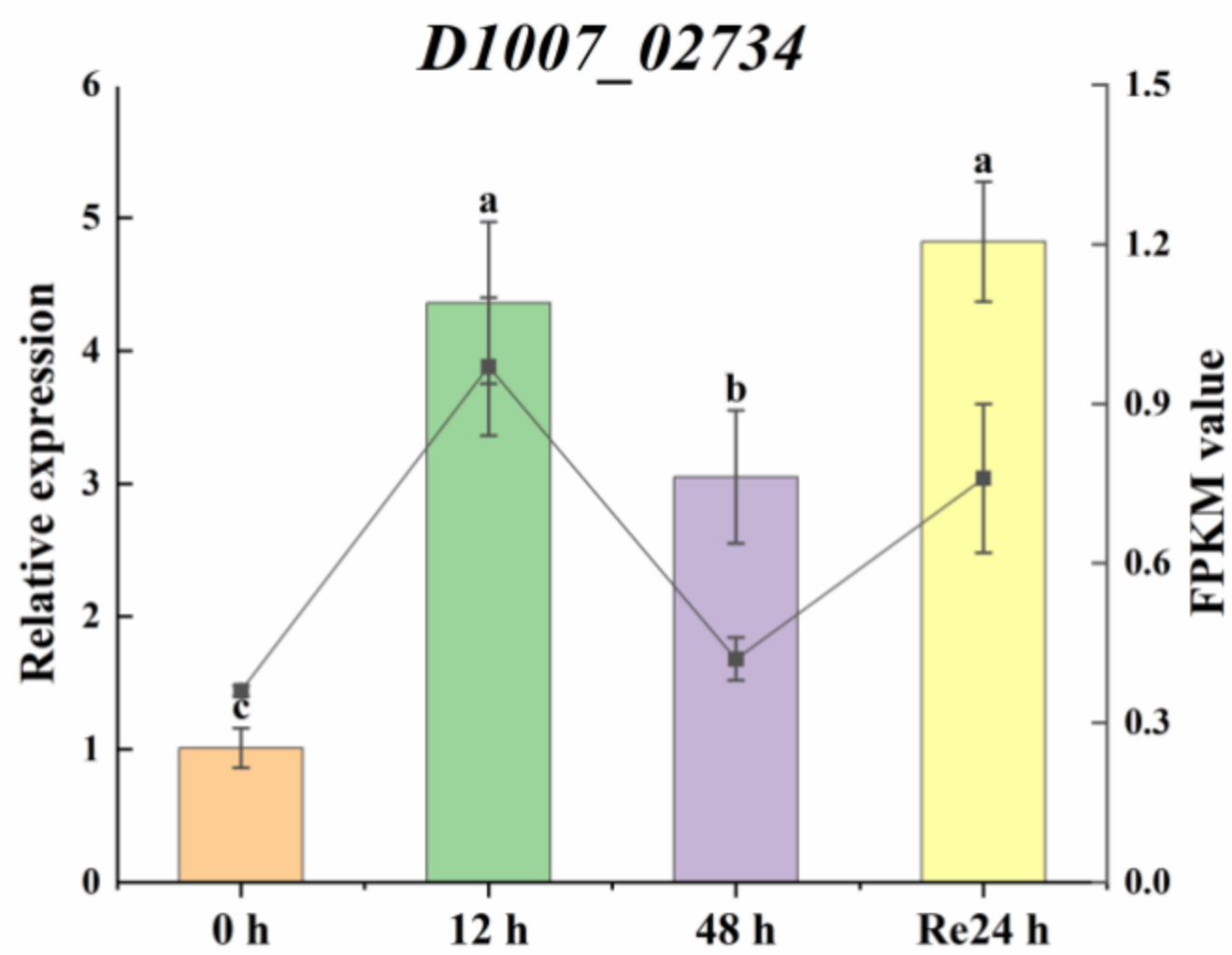

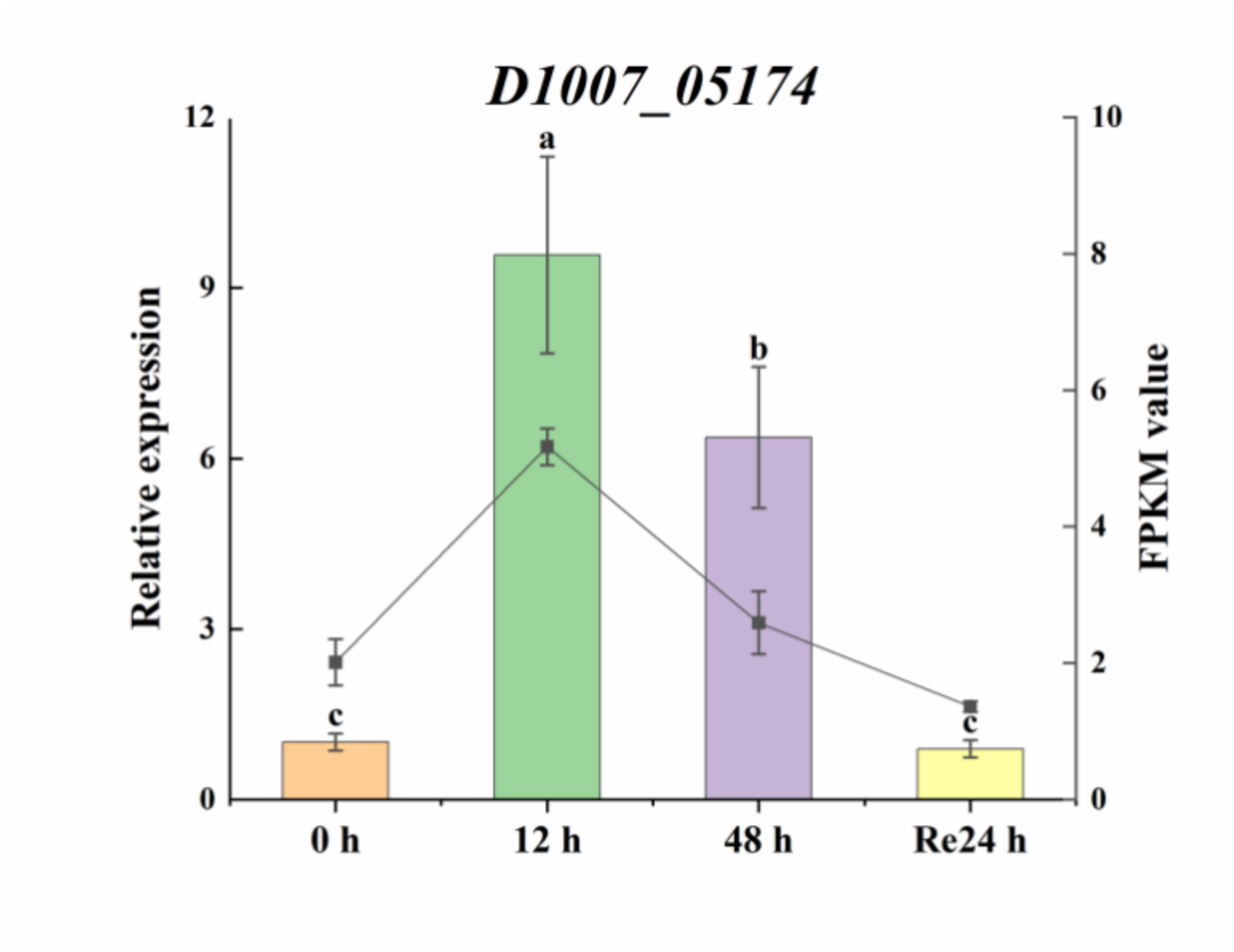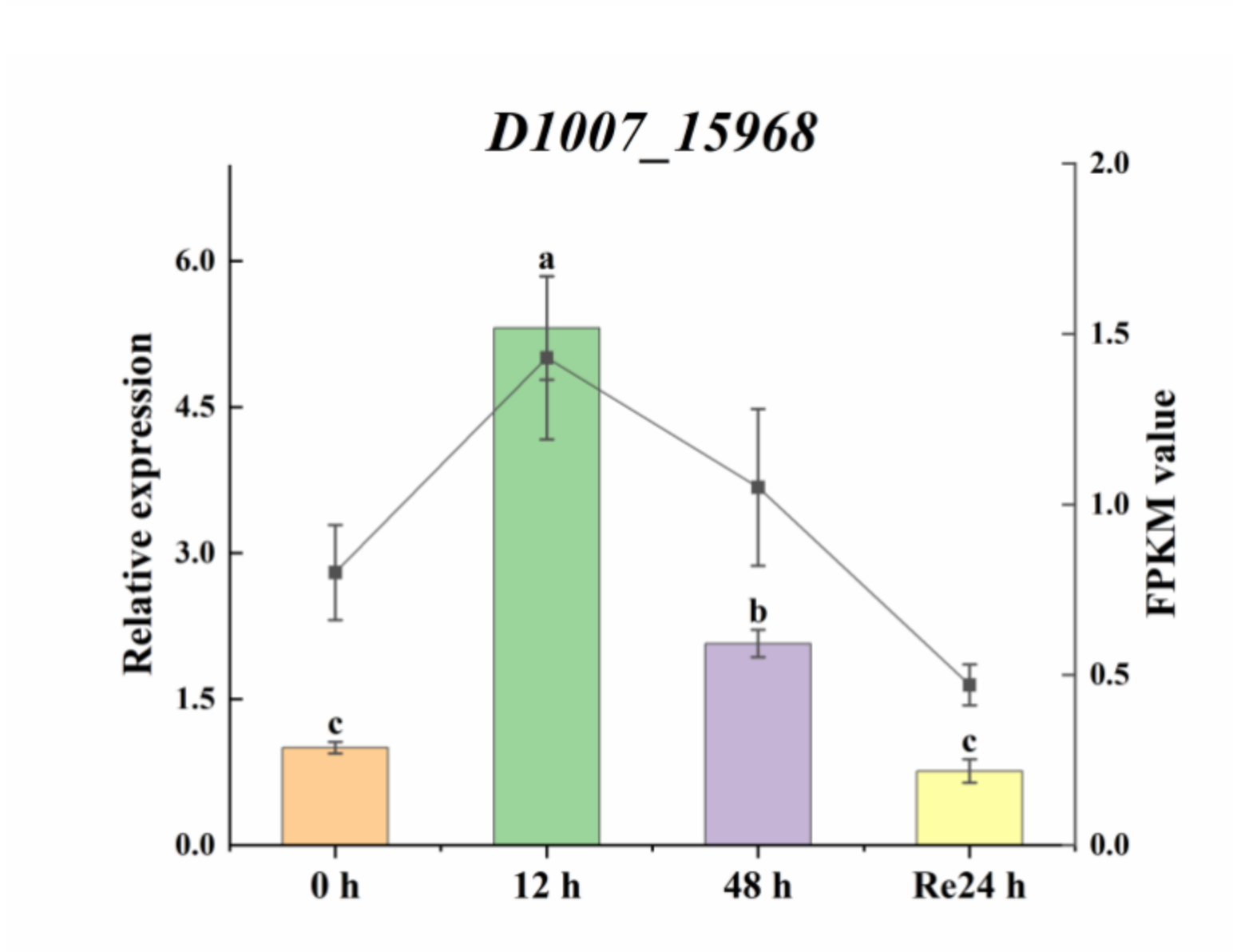

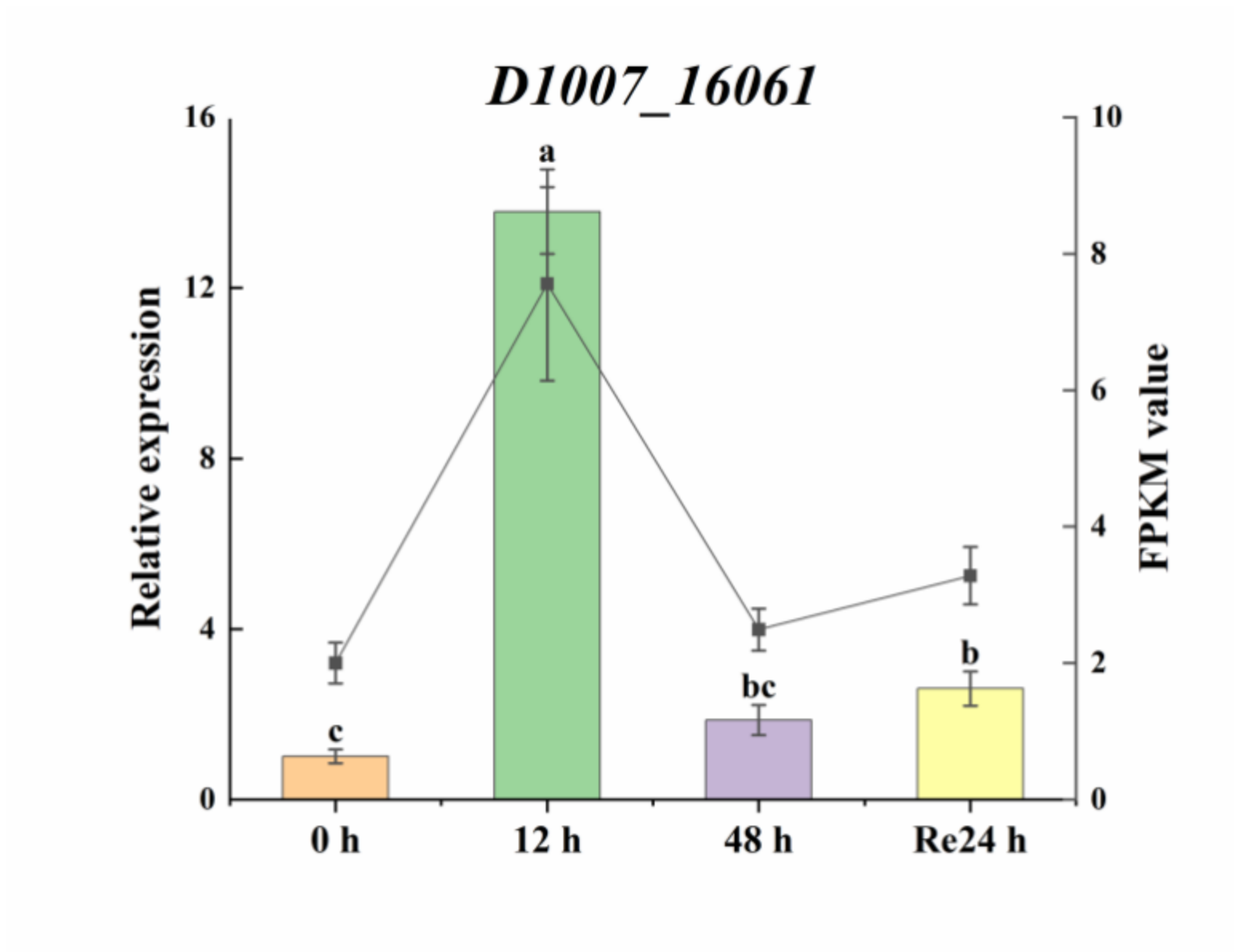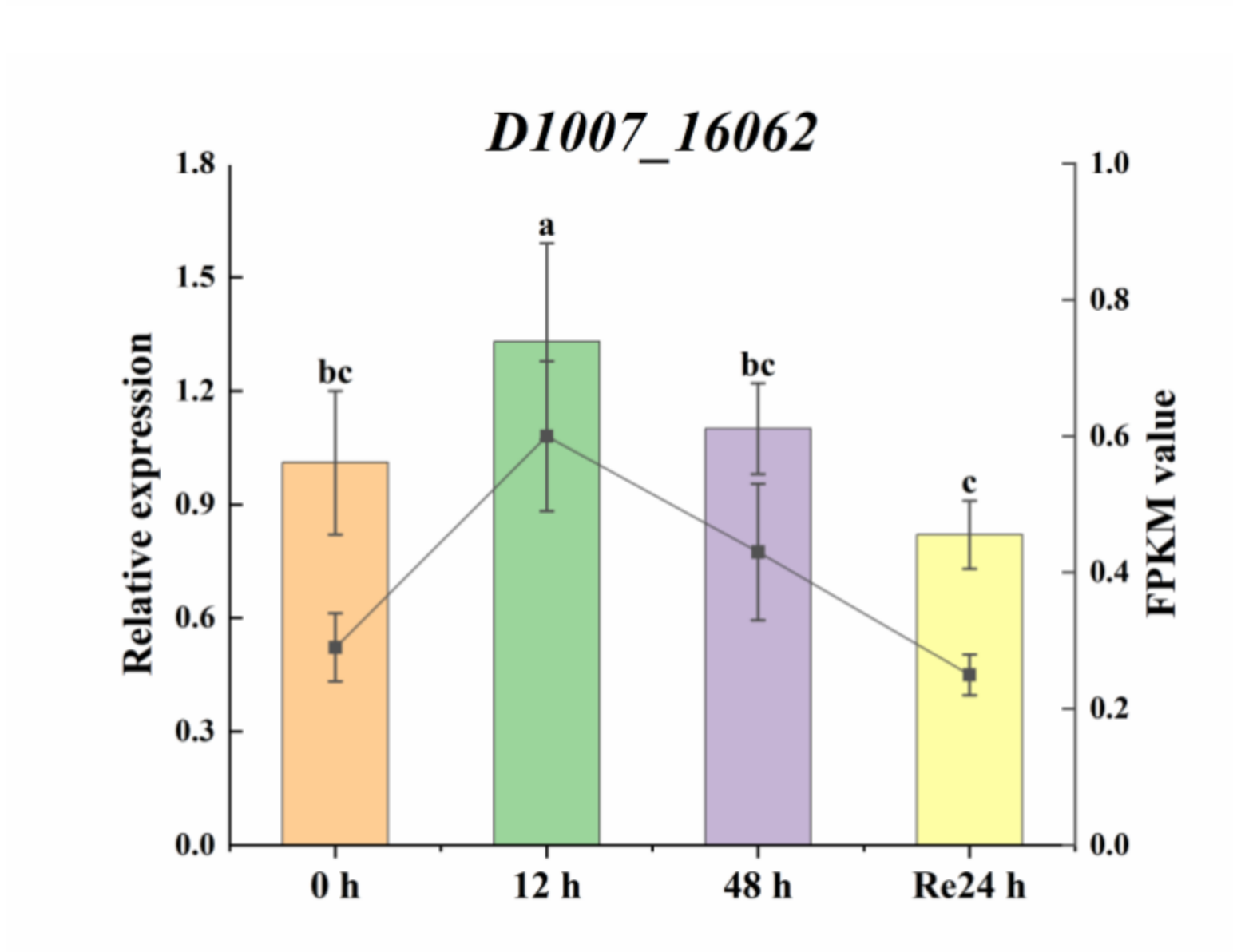

***D1007\_18040***

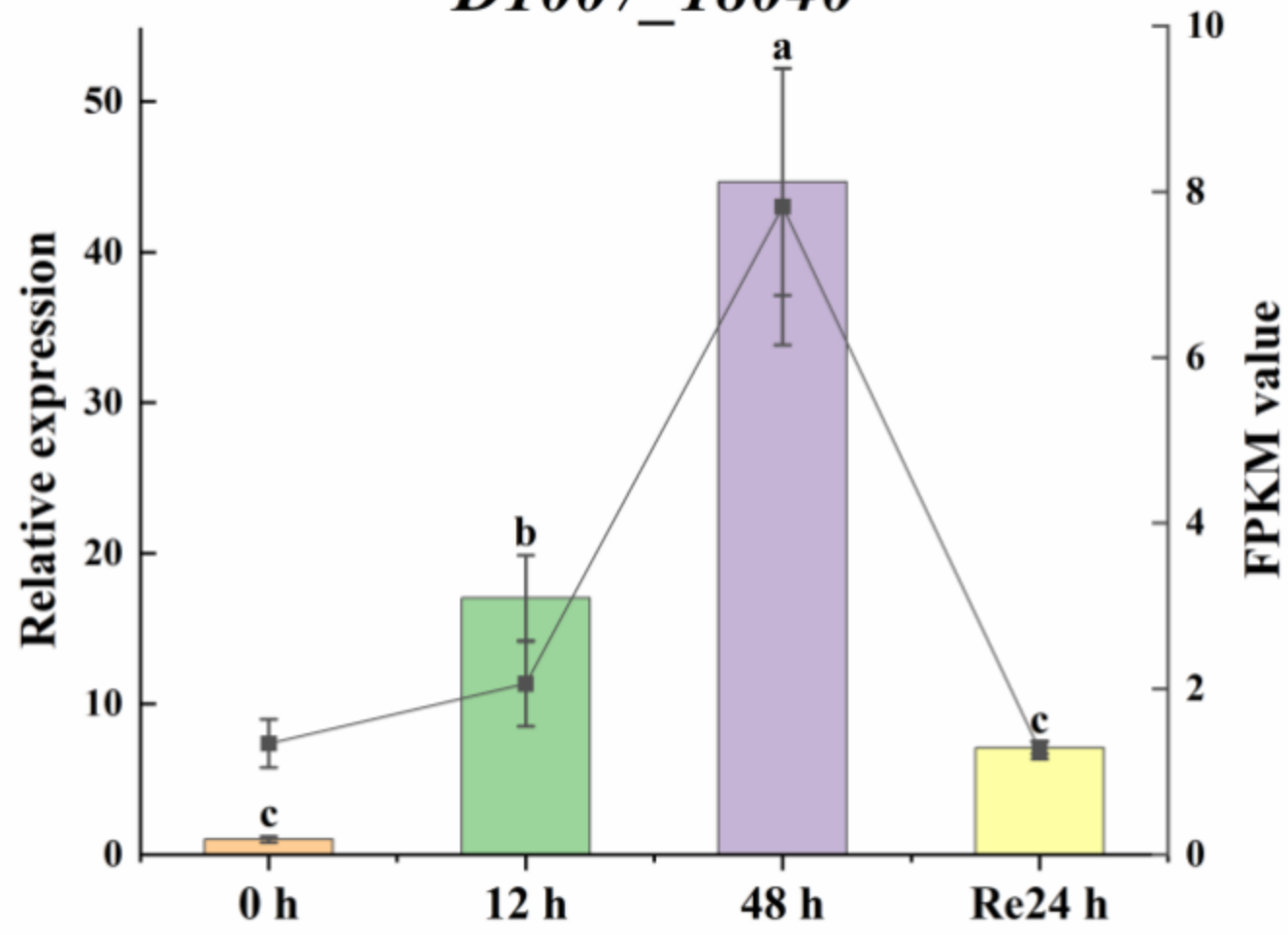

***D1007\_24605***

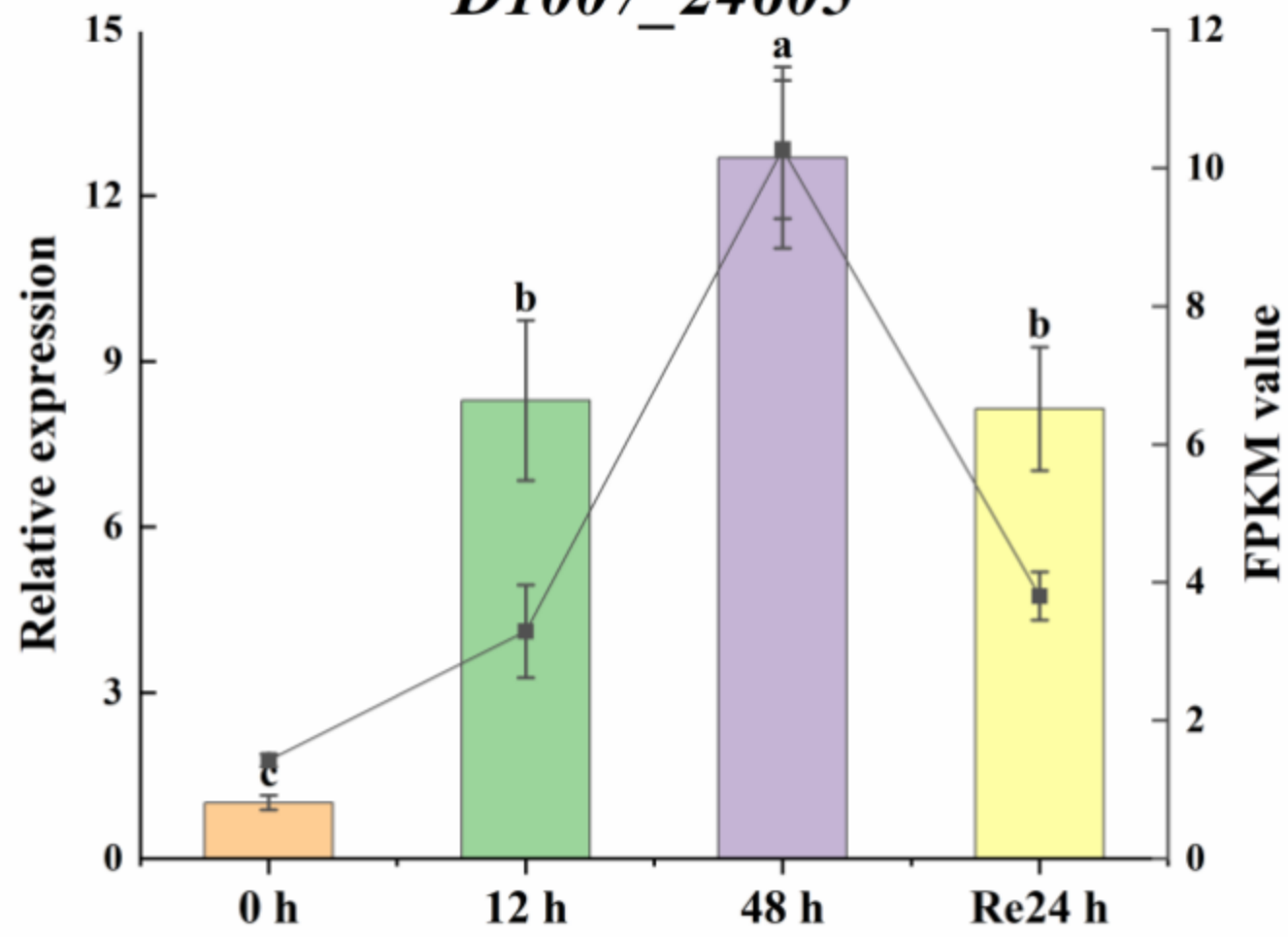

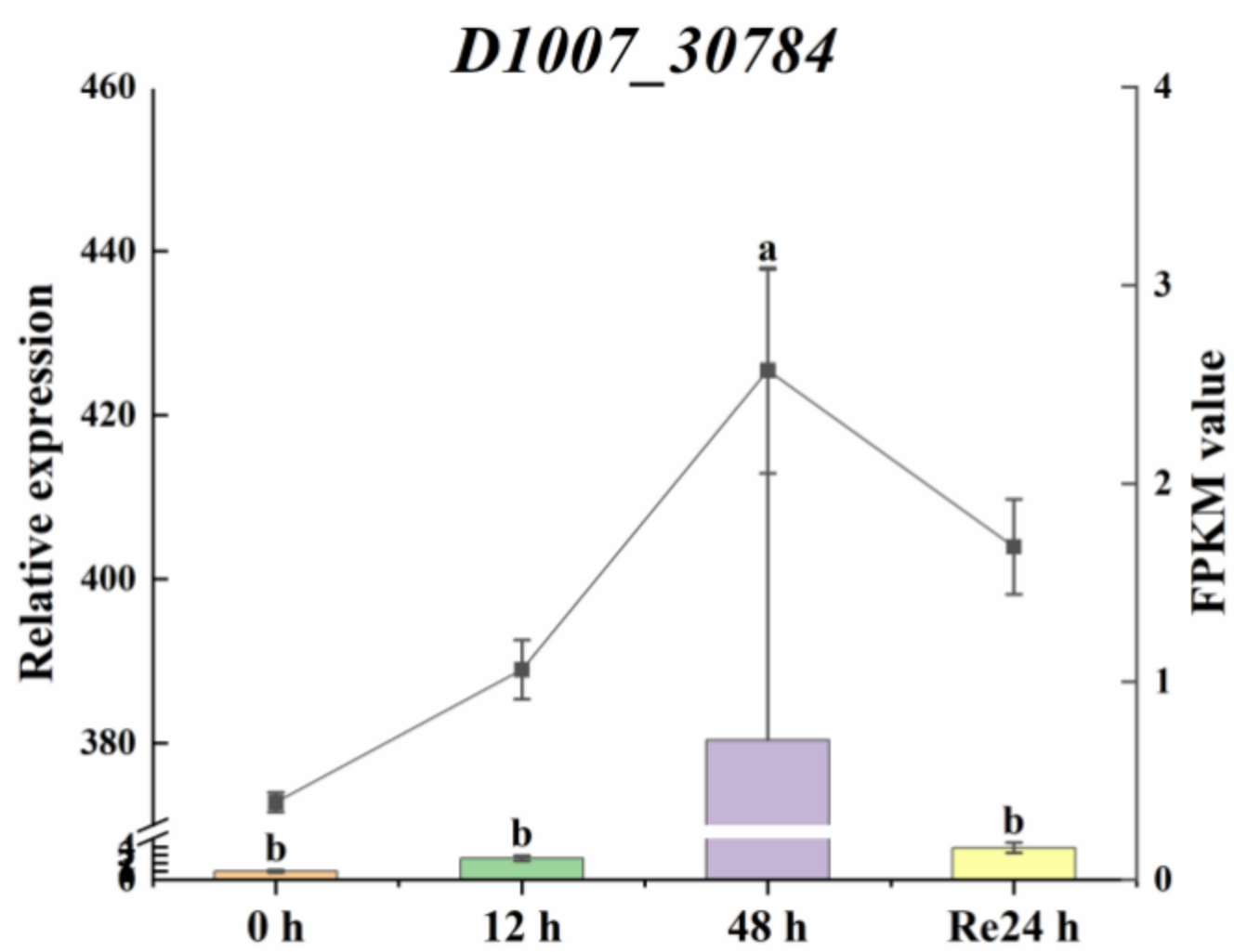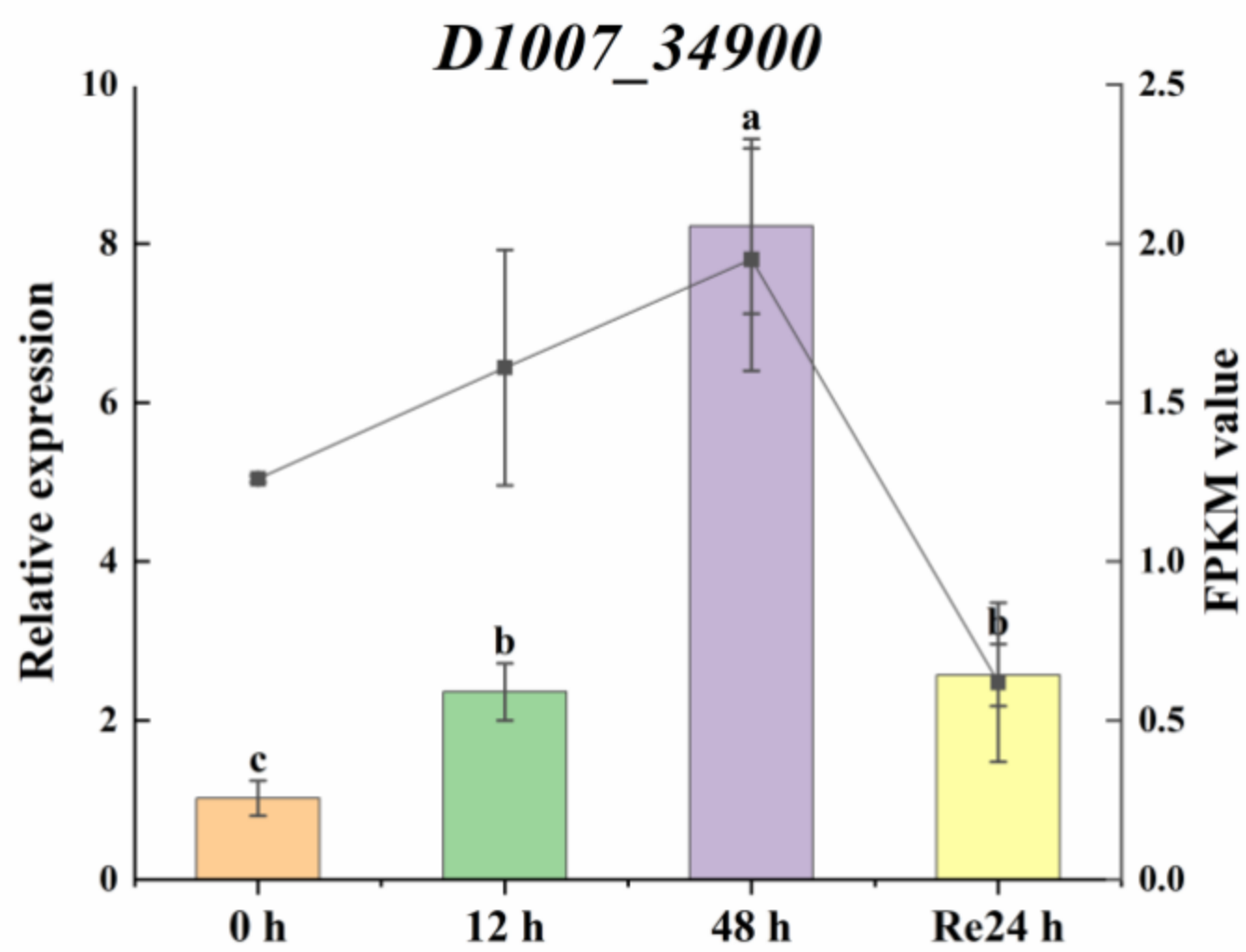

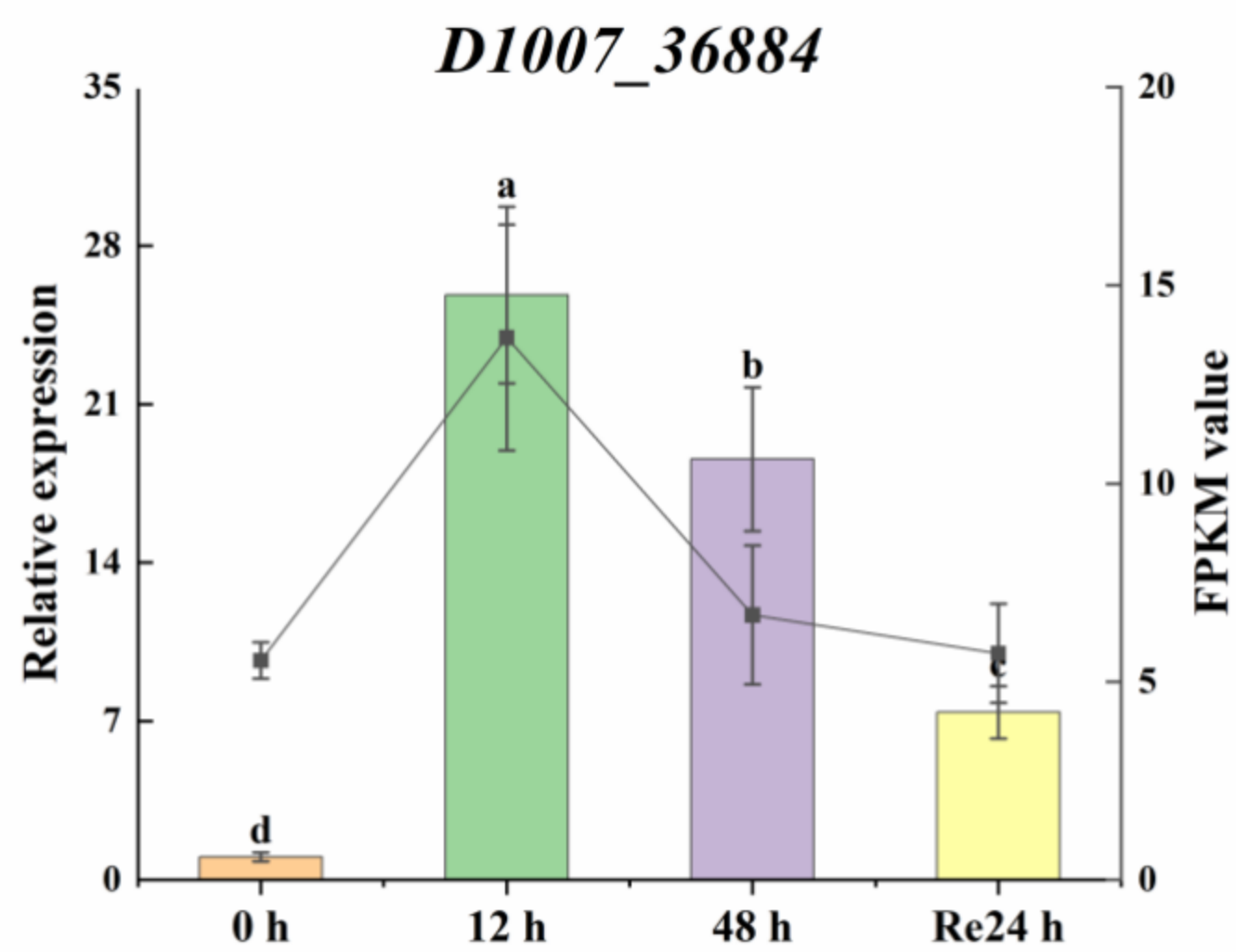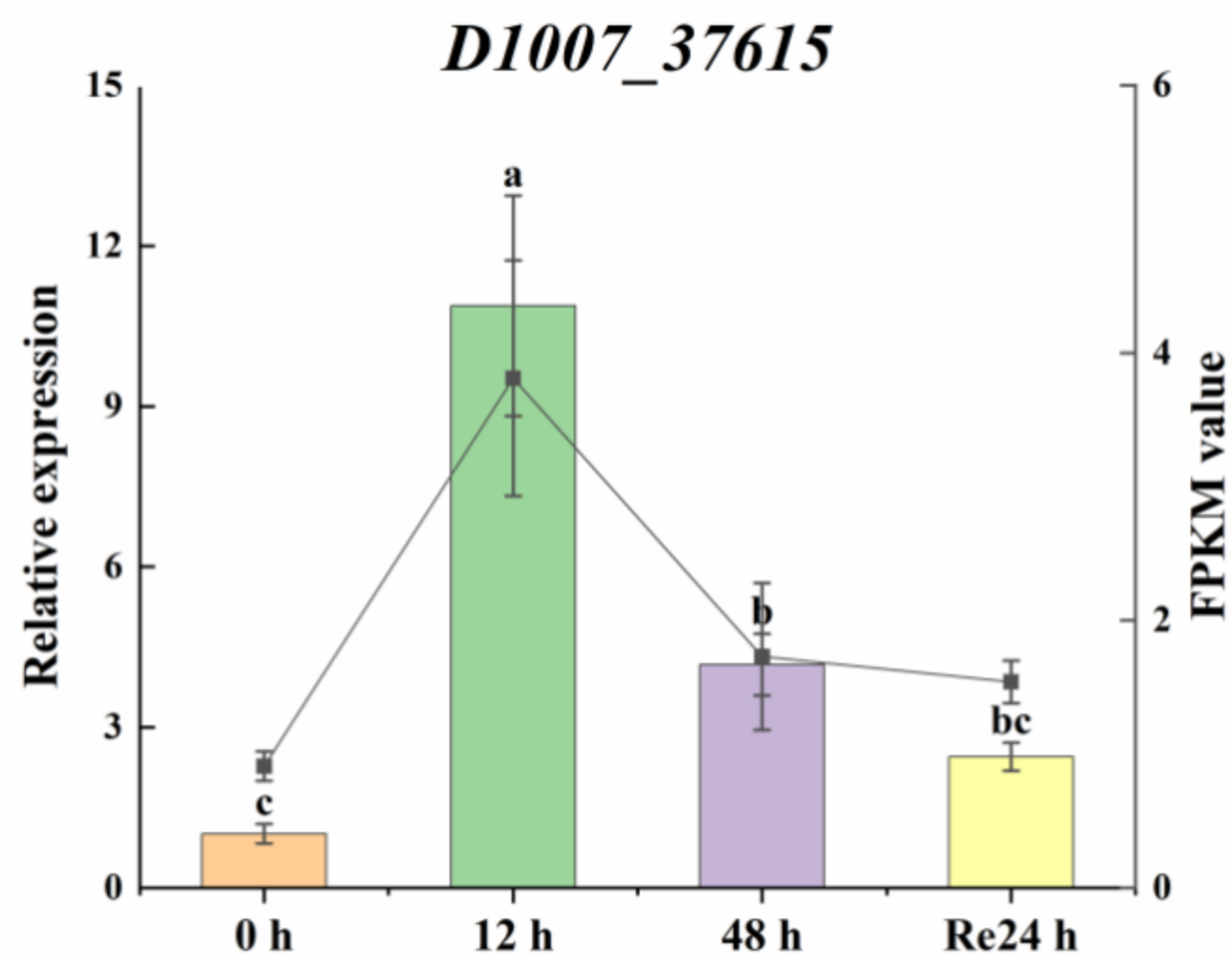

***D1007\_39096***

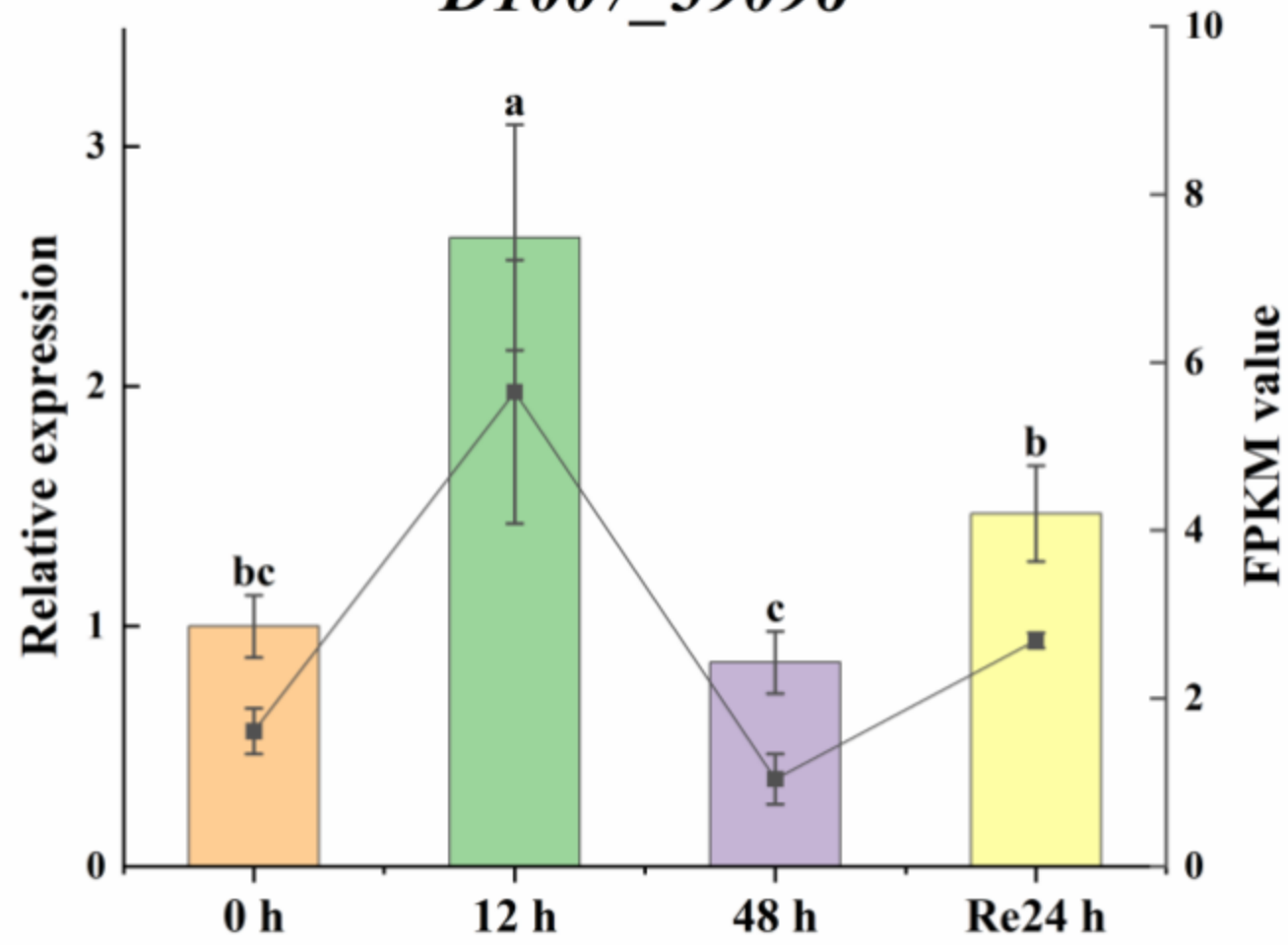

***D1007\_41116***

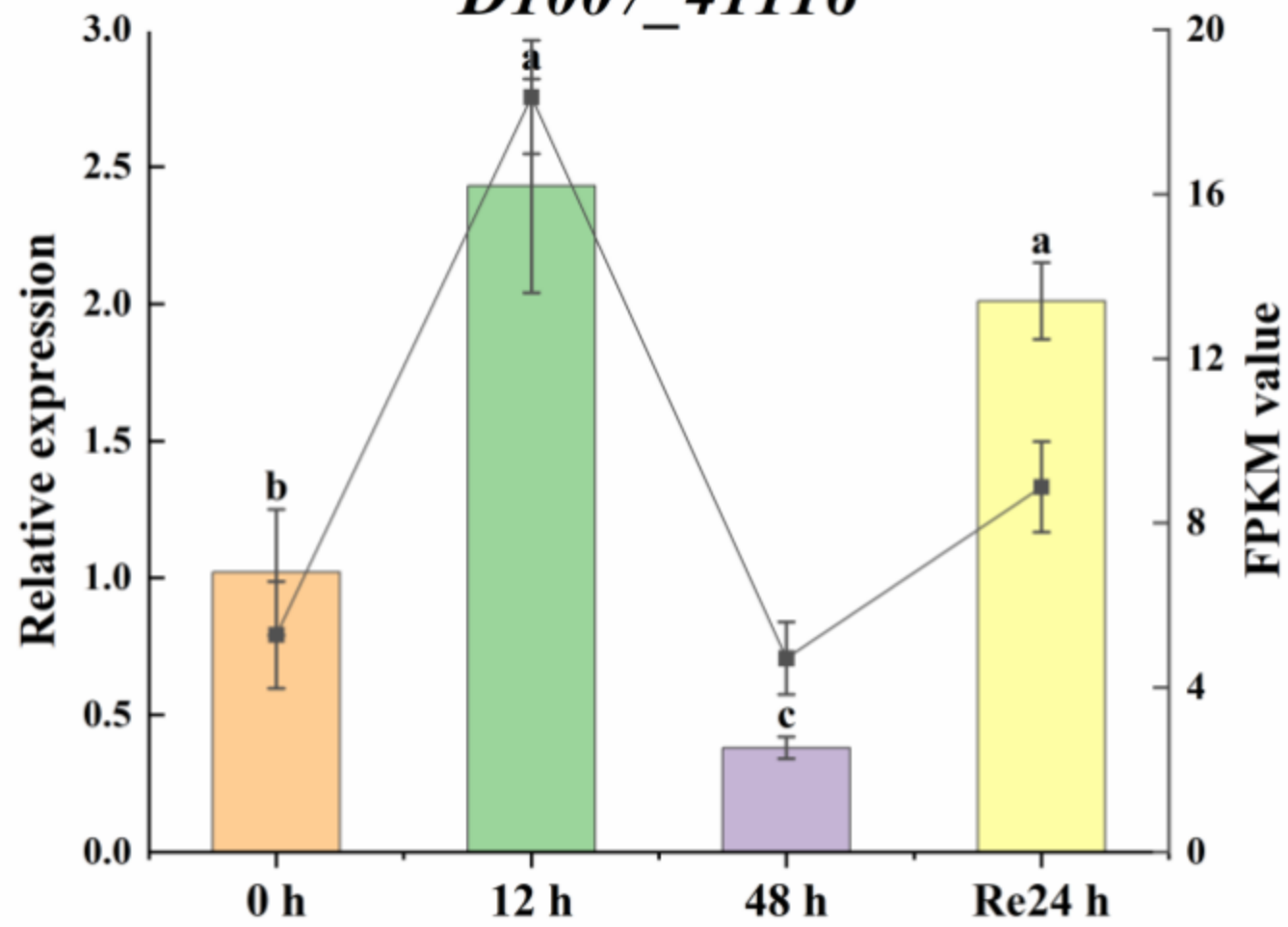

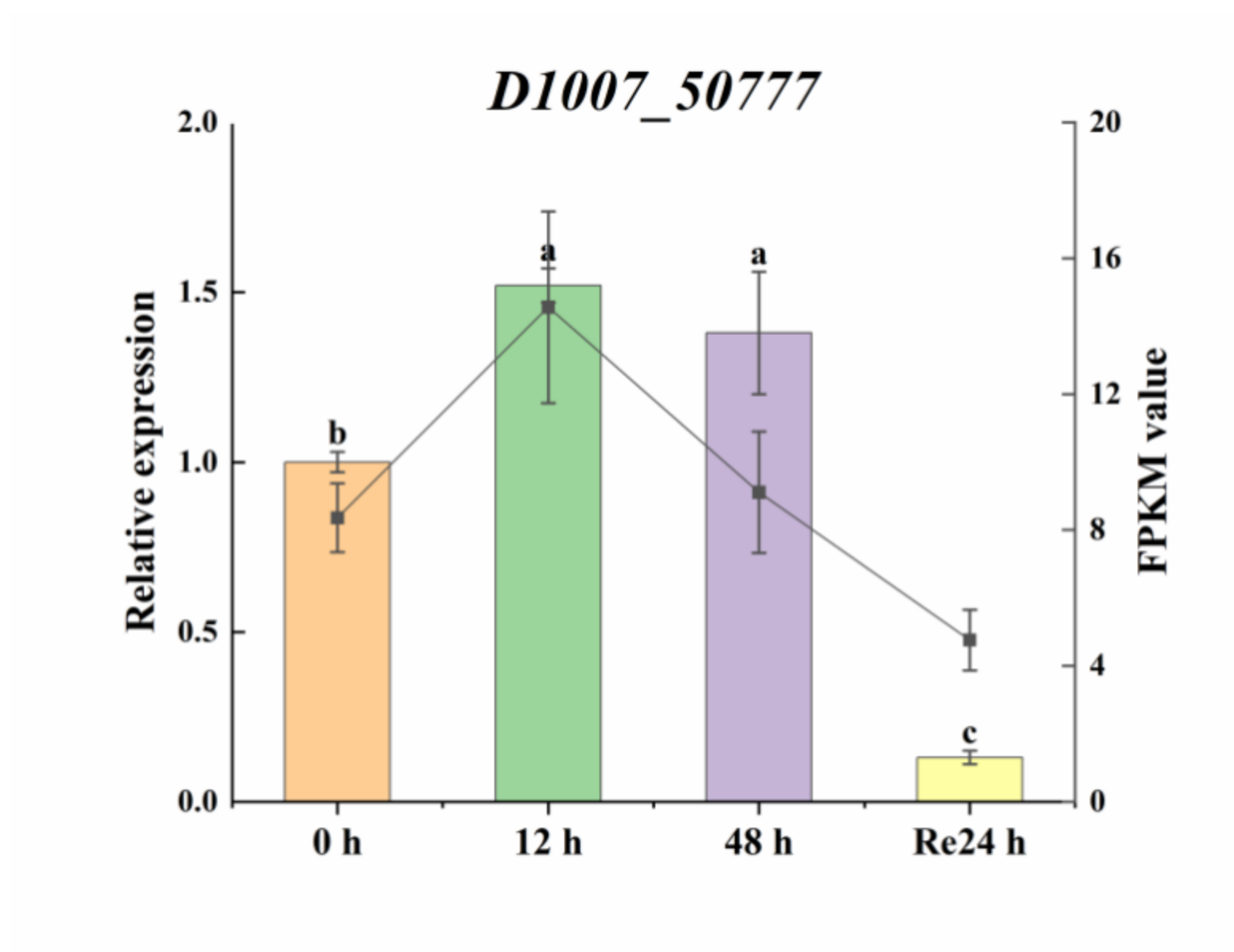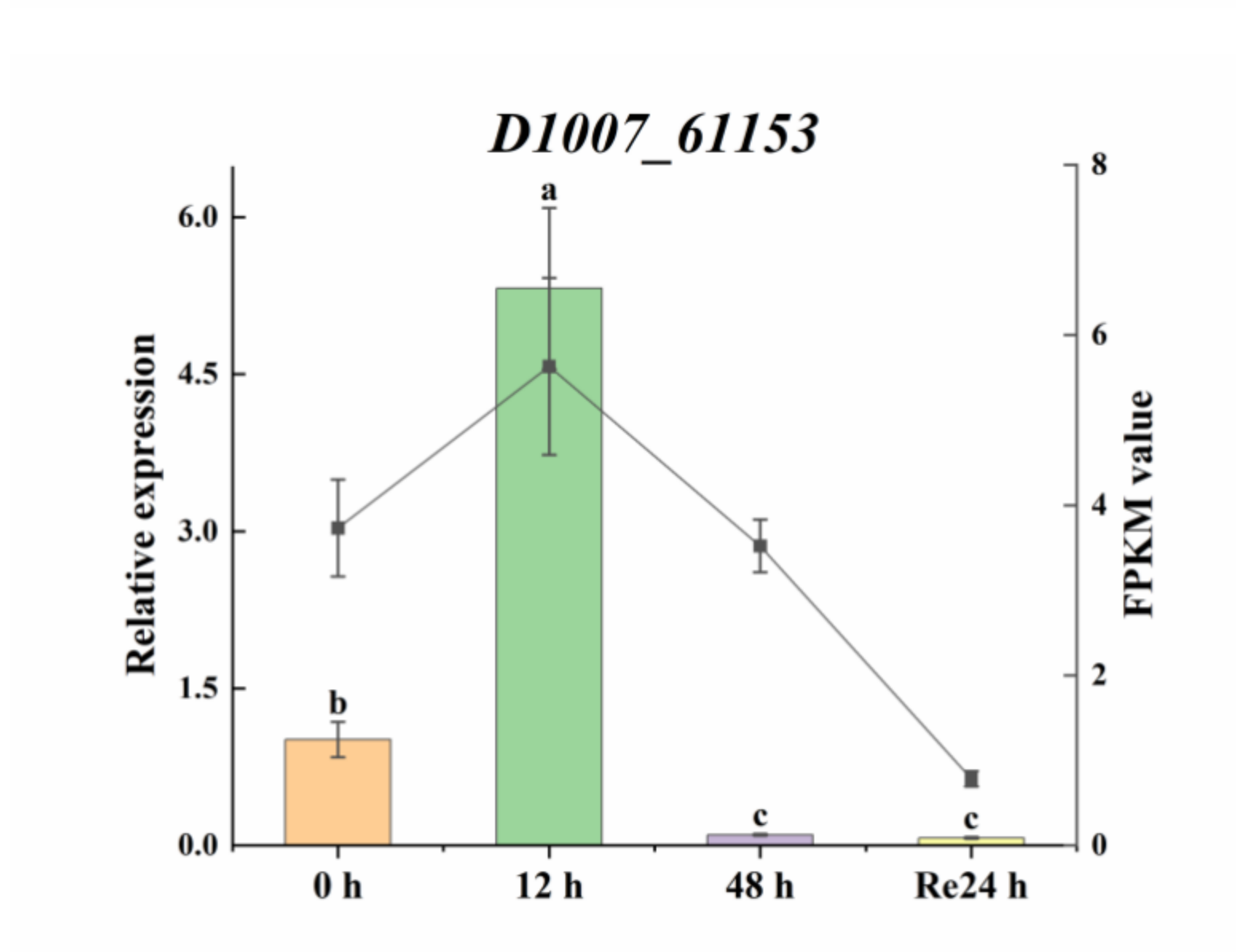

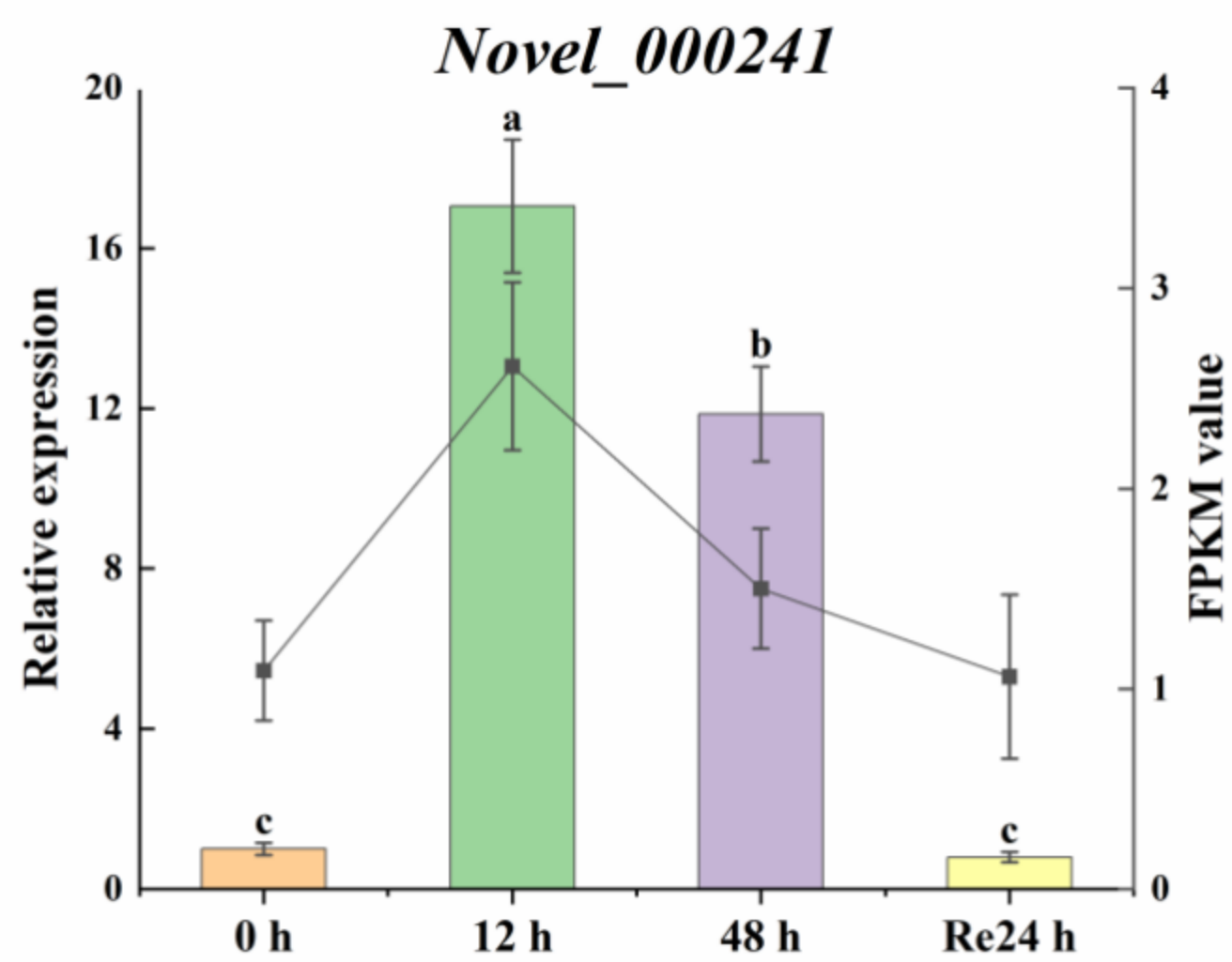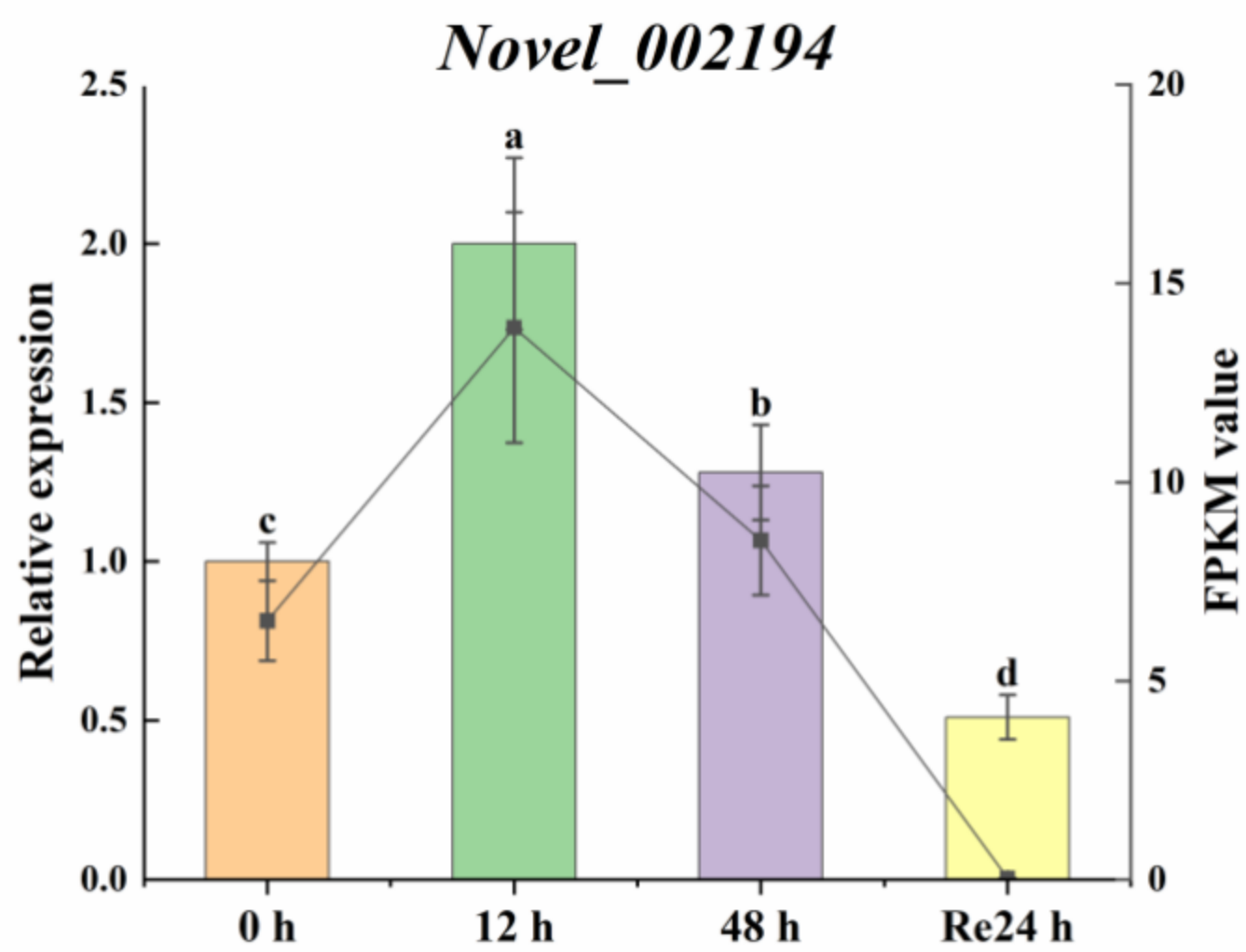

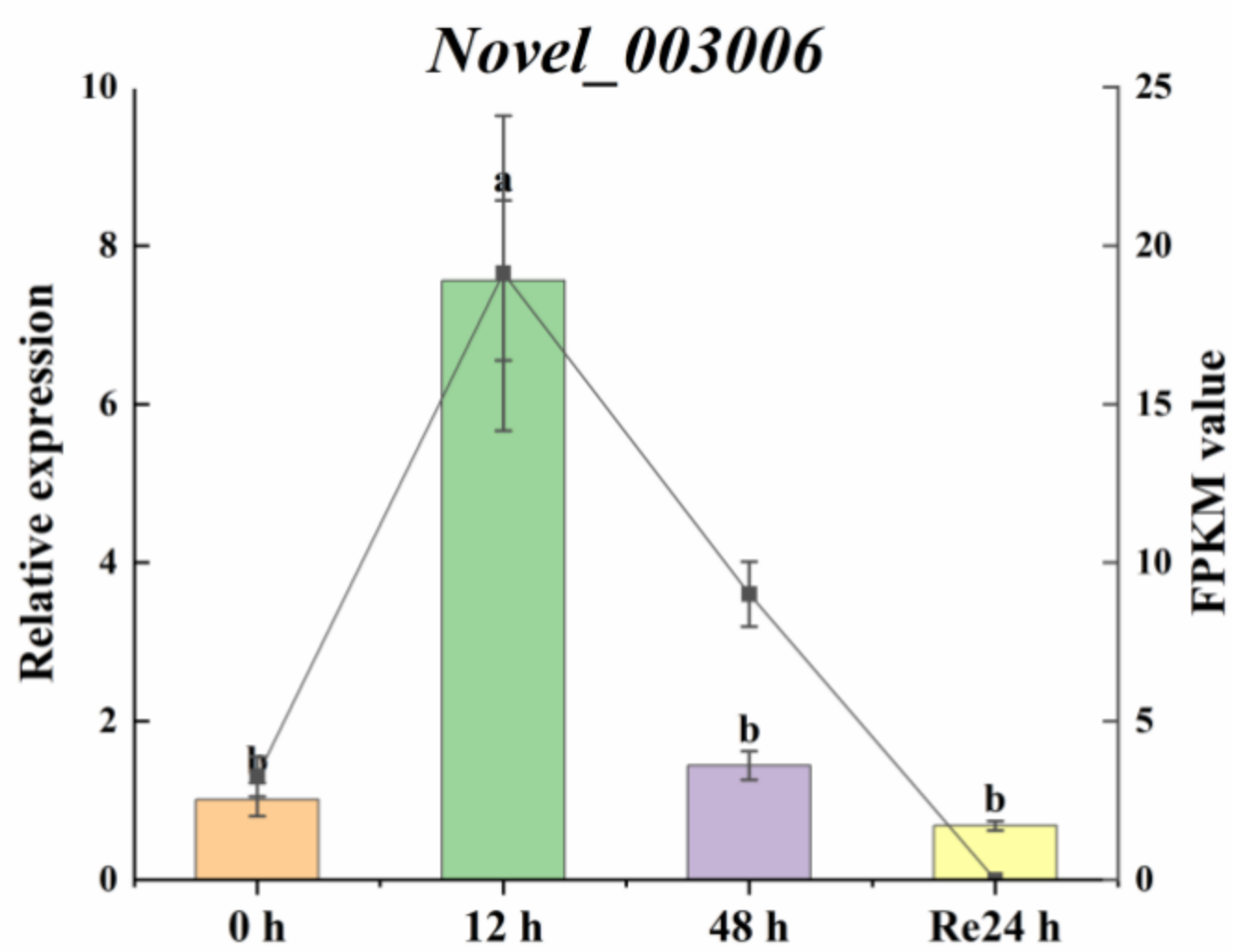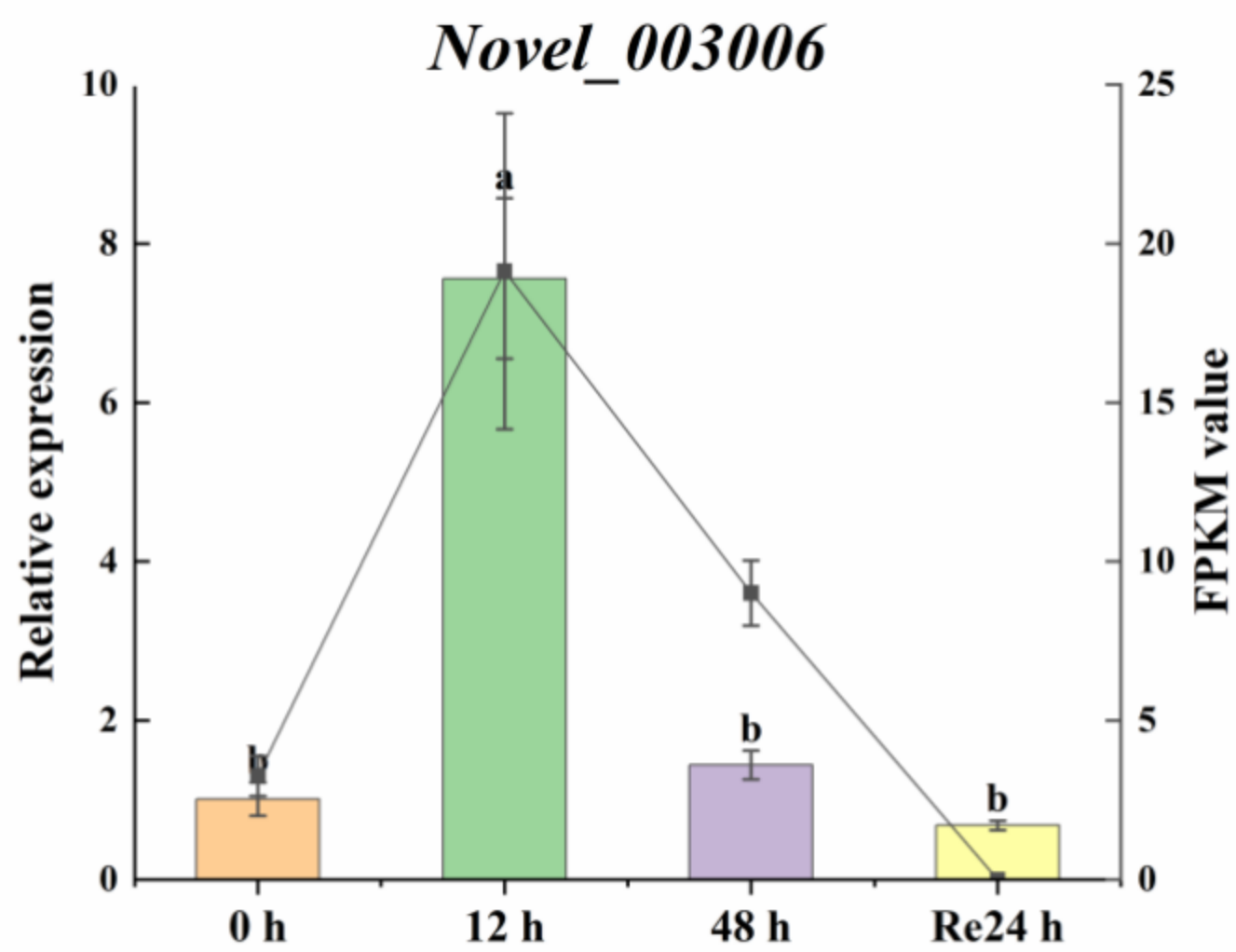

### *Novel\_003151*

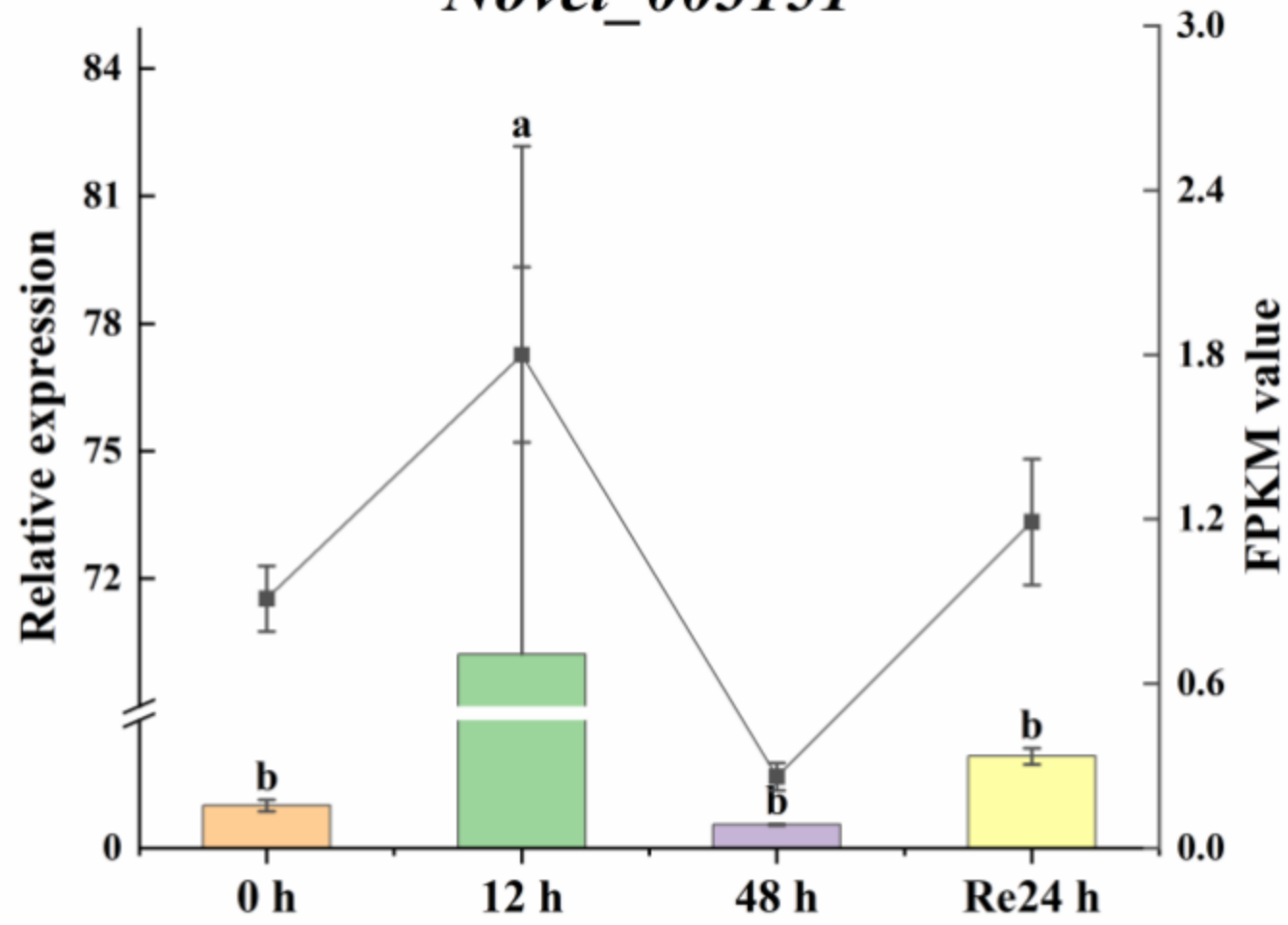

### *Novel\_006628*

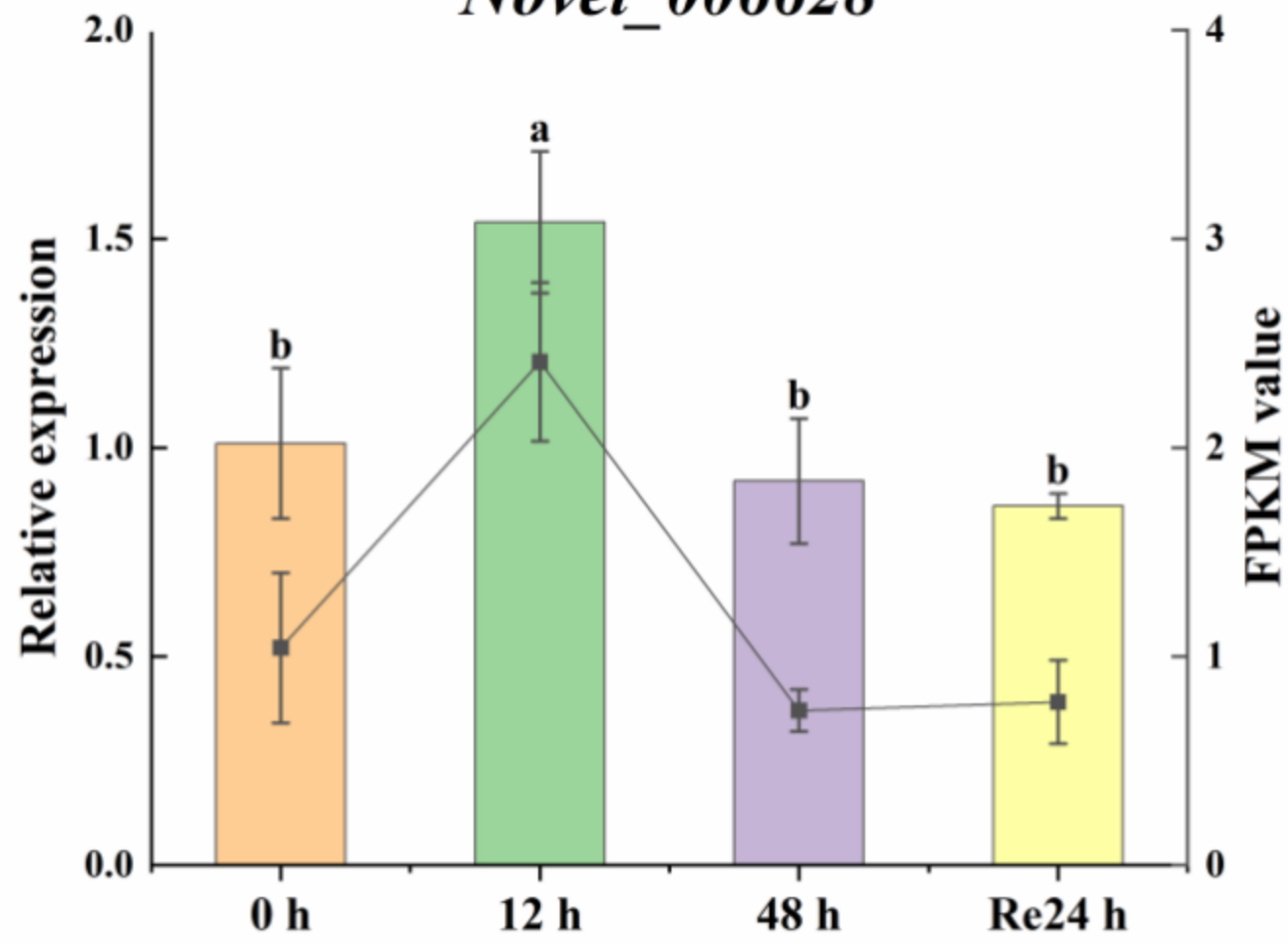

### *Novel\_006698*

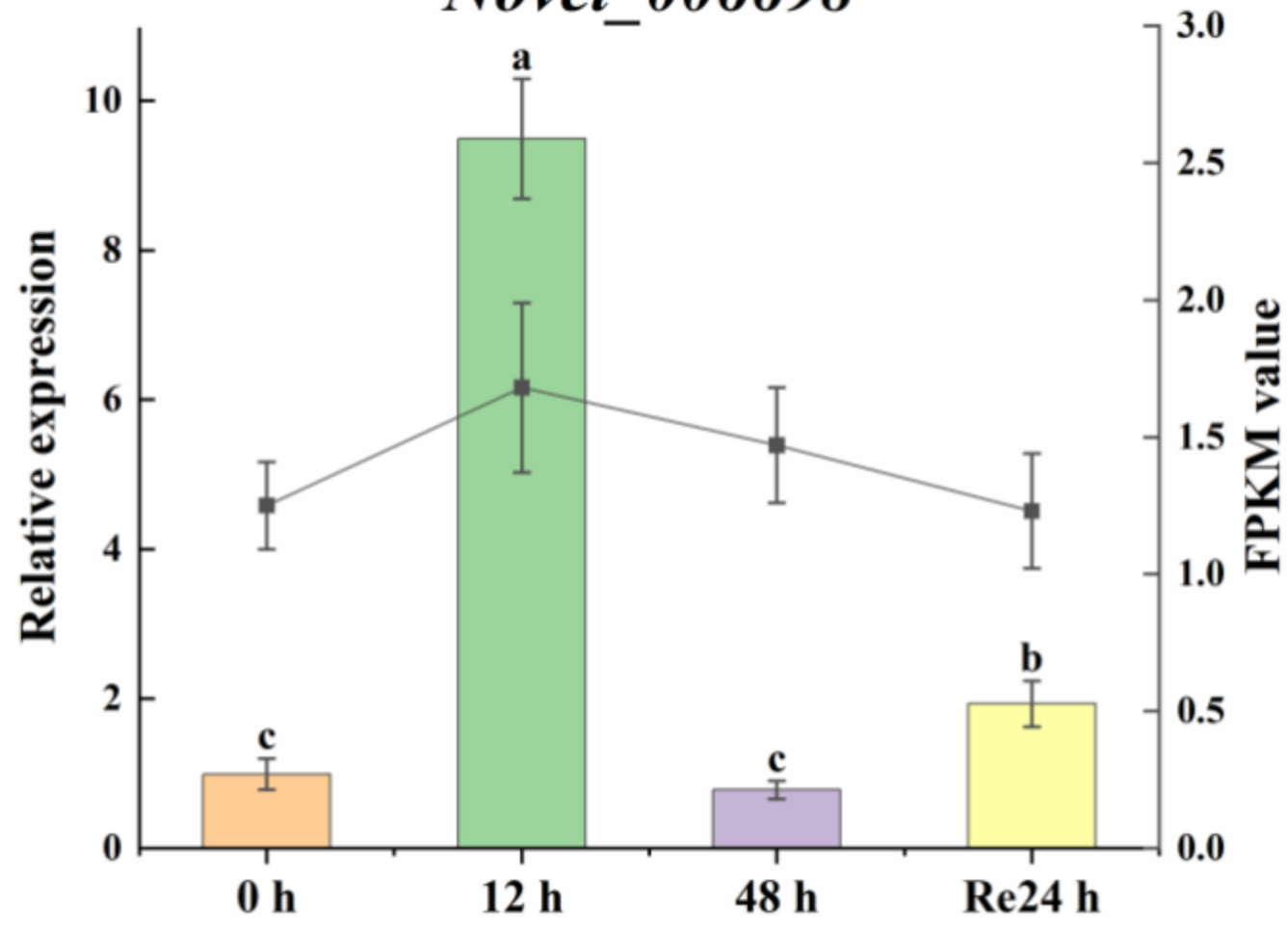

### *Novel\_008488*

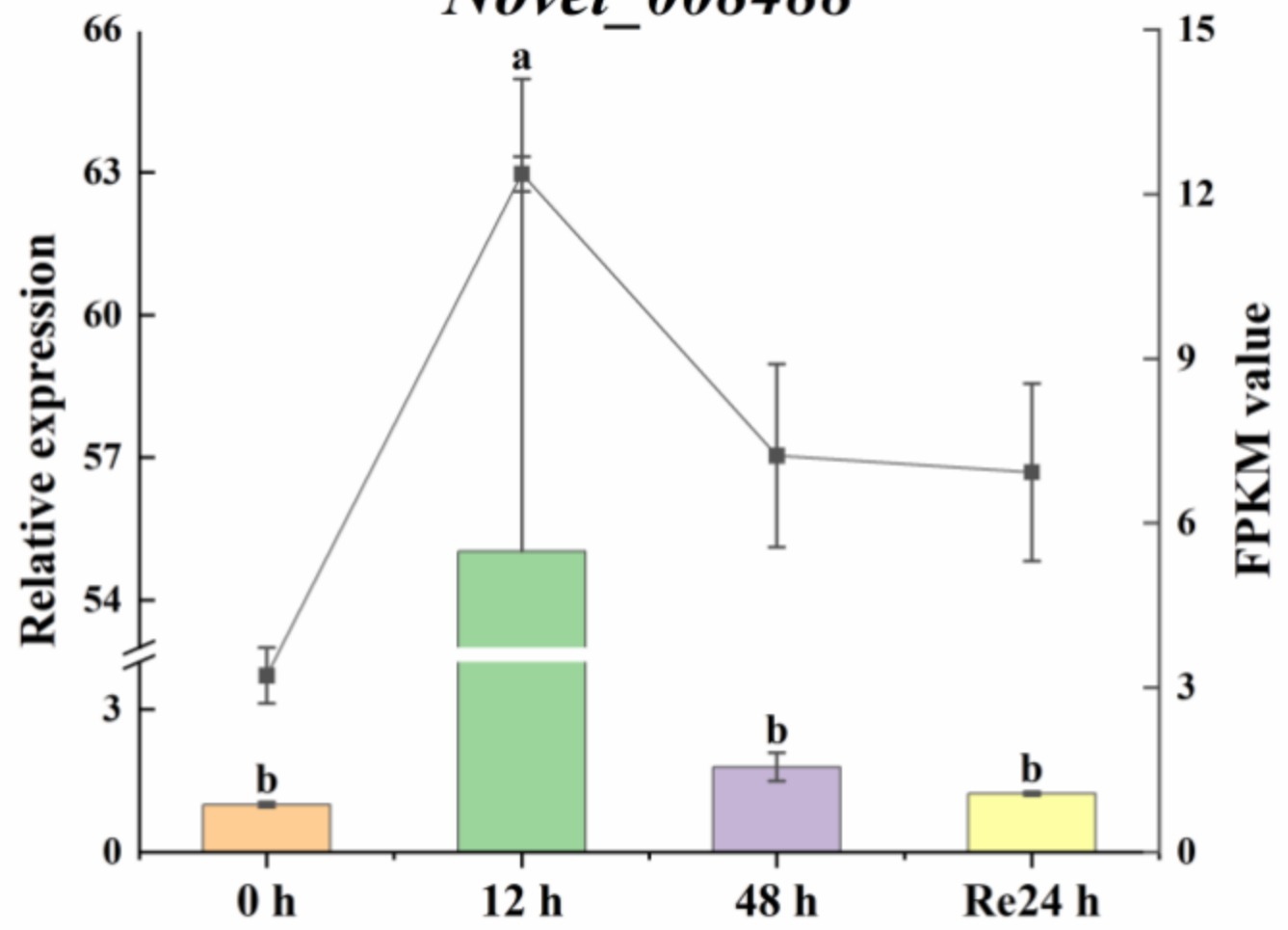

Supplement: Supplementary file 7 — Supplementary Material 7 [file 12864_2026_12838_MOESM7_ESM.pdf]
